# *Trans*‐A_2_B_2_‐Type Metalloporphyrin‐Based Donor–Acceptor Covalent Organic Frameworks for Efficient Photocatalytic CO_2_ Cycloaddition to Aziridines

**DOI:** 10.1002/advs.202513754

**Published:** 2025-11-12

**Authors:** Ji Xiong, Minghui Chen, Yunhao Xu, Yaqing Feng, Jian Song, Bao Zhang

**Affiliations:** ^1^ School of Chemical Engineering & Technology Tianjin University Tianjin 300350 P. R. China; ^2^ Tianjin Collaborative Innovation Center of Chemical Science and Engineering Tianjin 300072 P. R. China

**Keywords:** CO_2_ cycloaddition reaction, covalent organic frameworks, D‐A structure, photocatalysis, *trans*‐A_2_B_2_‐type metallized porphyrin

## Abstract

The sunlight‐driven CO_2_ cycloaddition to aziridines represents a promising strategy for CO_2_ resource utilization, offering a green alternative to conventional thermally driven fixation approach that typically requires high temperatures and/or elevated pressures. Inspired by the exceptional light‐absorption properties of porphyrin derivatives and the enhanced charge separation afforded by a donor‐acceptor (D‐A) configuration, two porphyrin‐based D‐A type covalent organic frameworks (COFs), including metal‐free *m*‐DBPA‐COF and metallized *m*‐NiDBPA‐COF are synthesized through acid‐catalyzed Schiff base reaction between electron donor (tris(4‐aminophenyl)amine) and electron acceptor (*trans*‐A_2_B_2_‐type *m*‐DBP‐CHO or *m*‐NiDBP‐CHO) units. The incorporation of *trans*‐A_2_B_2_‐type metalloporphyrin markedly enhances the transfer and separation of photoinduced charge carriers through ligand‐to‐metal charge transfer (LMCT) and the electron push‐pull characteristic inherent in D‐A configurations. Moreover, the incorporated Ni ions provide Lewis acidic sites that facilitate substrate interactions. Encouragingly, under visible light‐assisted and mild conditions (1 atm CO_2_ with no heating required), *m*‐NiDBPA‐COF exhibited remarkable photocatalytic performance, achieving a reaction rate of 4.13 mol mol^−1^ h^−1^, which is comparable to that of most thermal catalysts in catalyzing the CO_2_ cycloaddition to aziridines. Overall, the study not only provides a guide for the design of porphyrin‐based COF photocatalysts, but also offers a green route to address the CO_2_‐related resource utilization issues.

## Graphical abstract

Two novel *trans*‐A_2_B_2_‐type porphyrin‐based D‐A type covalent organic frameworks (COFs), including metal‐free *m*‐DBPA‐COF and metallized *m*‐NiDBPA‐COF, have been successfully synthesized and under visible light‐assisted and mild conditions (1 atm CO_2_ with no heating required), *m*‐NiDBPA‐COF exhibits remarkable photocatalytic performance, achieving a reaction rate comparable to that of most thermal catalysts in catalyzing the CO_2_ cycloaddition with aziridines.

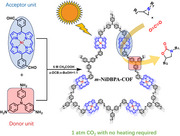

## 1 Introduction

The increasing use of conventional energy sources has led to the continuous growth of carbon dioxide (CO_2_) concentration in the atmosphere, resulting in global warming.^[^ ^1^, ^2^, ^3^ ^]^ To mitigate the greenhouse effect, capture, storage, and utilization of CO_2_ have been undertaken.^[^ ^4^, ^5^ ^]^ Nevertheless, it is worth noting that CO_2_ is an abundant, non‐toxic, and renewable C1 source.^[^ ^6^, ^7^, ^8^, ^9^ ^]^ Green chemical conversion of CO_2_ not only efficiently resolves excess emissions but also yields various high‐value products.^[^ ^10^, ^11^, ^12^ ^]^ Among environmentally benign CO_2_ conversion reactions, the cycloaddition with aziridines to produce oxazolidinones represents a promising pathway due to its 100% atom economy.^[^ ^13^ ^]^ Moreover, oxazolidinones have diverse applications as chiral auxiliaries and antibacterial pharmaceuticals,^[^ ^14^, ^15^, ^16^ ^]^ attracting considerable attention. However, CO_2_ is thermodynamically and kinetically stable due to its strong C═O bond (805 kJ mol^−1^), resulting in low reactivity.^[^ ^17^ ^]^ Various homogeneous catalysts, such as alkali metal halides,^[^ ^18^ ^]^ ionic liquids,^[^ ^19^ ^]^ and metal complexes,^[^ ^20^, ^21^ ^]^ have been employed for the cycloaddition of CO_2_ with aziridines. Nevertheless, the major drawback is that these homogeneous catalysts are difficult to be separated and recovered, making the reaction process environmentally unfriendly and cost‐ineffective. In contrast, heterogeneous catalysts with excellent catalytic activity and recyclability are crucial for industrial production. However, only a few heterogeneous catalysts have been reported for this reaction,^[^ ^14^, ^15^, ^22^, ^23^, ^24^, ^25^ ^]^ and in most cases, high CO_2_ pressure and/or high temperature conditions are required. Therefore, the rational design and synthesis of efficient heterogeneous catalysts for catalyzing the cycloaddition of CO_2_ with aziridines under mild conditions is of sufficient significance.

Porphyrins, a class of conjugated π‐electron aza‐macrocycles, exhibit significant potential due to their unique physicochemical properties, including extensive light absorption in the visible spectrum^[^ ^26^ ^]^ and a pronounced affinity to CO_2_.^[^ ^27^ ^]^ Metal ions can be inserted into porphyrin rings through coordination with the four nitrogen atoms, thereby forming efficient metal‐porphyrin catalytic centers.^[^ ^28^ ^]^ These central metal ions can function as Lewis acidic sites, facilitating substrate interactions and promoting the directional migration of photogenerated excitons.^[^ ^29^, ^30^ ^]^ Homogeneous porphyrin catalysts have demonstrated effectiveness in catalyzing CO_2_ cycloaddition to aziridines.^[^ ^31^, ^32^, ^33^ ^]^

Covalent organic frameworks (COFs), composed of topologically repeating units interconnected by covalent bonds, are increasingly significant in photovoltaic and optoelectronic applications due to their remarkable features, such as aligned pore architectures and customizable functionalities.^[^ ^34^ ^]^ Integrating molecular porphyrin units into crystalline COFs can lead to the functional and ordered networks with well‐defined pores, high surface areas, robust CO_2_ adsorption capabilities, and excellent recyclability in catalytic applications.^[^ ^35^, ^36^, ^37^, ^38^ ^]^ Moreover, employing a donor‐acceptor (D‐A) configuration enables precise tuning of the bandgap of porphyrin‐based COFs and enhances the efficiency of electron‐hole separation.^[^ ^39^, ^40^, ^41^, ^42^ ^]^ However, to date, most porphyrinic COFs have been constructed using symmetrical (A_4_) porphyrin building units.^[^ ^36^, ^43^, ^44^ ^]^ In contrast, COFs based on *trans*‐A_2_B_2_‐type porphyrin building blocks, where the “A” substituents are positioned at the 5,15‐positions and the “B” substituents at the 10,20‐positions, have rarely been reported,^[^ ^45^ ^]^ likely due to the synthetic challenges associated with these molecules. Compared with A_4_ porphyrin building units, *trans*‐A_2_B_2_‐type porphyrin building blocks enable the formation of porphyrin‐based COFs with distinct topological architectures, which are inherently linked to their functional properties.

Herein, two *trans*‐A_2_B_2_‐type porphyrin monomers, 5,15‐di(3‐benzaldehyde)porphyrin (*m*‐DBP‐CHO) and [5,15‐di(3‐benzaldehyde)porphyrinato]nickel(II) (*m*‐NiDBP‐CHO), were successfully synthesized and utilized for constructing two isostructural porphyrinic COFs, namely *m*‐DBPA‐COF and *m*‐NiDBPA‐COF, which were constructed from a conjugated electron donor, tris(4‐aminophenyl)amine (TAPA), and the electron‐deficient moiety, *m*‐DBP‐CHO or *m*‐NiDBP‐CHO, via acid‐catalyzed Schiff‐base reactions. The ligand‐to‐metal charge transfer (LMCT) occurred in metalloporphyrin, together with the donor‐acceptor (D‐A) type electronic push‐pull effect between the electron donor (TAPA) and the electron acceptor (metalloporphyrin), synergistically accelerated the transfer and separation of photo‐induced carriers. Moreover, the incorporated Ni ions provided Lewis acidic sites that facilitate substrate interactions. Encouragingly, under visible light‐assisted and mild conditions (1 atm CO_2_ with no heating required), *m*‐NiDBPA‐COF exhibited remarkable photocatalytic performance with the reaction rate reaching 4.13 mol mol^−1^ h^−1^, which was comparable to that of most thermal catalysts in catalyzing the CO_2_ cycloaddition to aziridines. This work is expected to guide the design of porphyrin‐based heterogeneous COF photocatalysts with well‐defined active metal sites and efficient exciton dissociation capabilities, which can be achieved by employing elaborately designed metalloporphyrin monomers and utilizing bottom‐up synthetic strategies.

## 2 Results and Discussion

### 2.1 Structure Characterization

**Figure 1:**
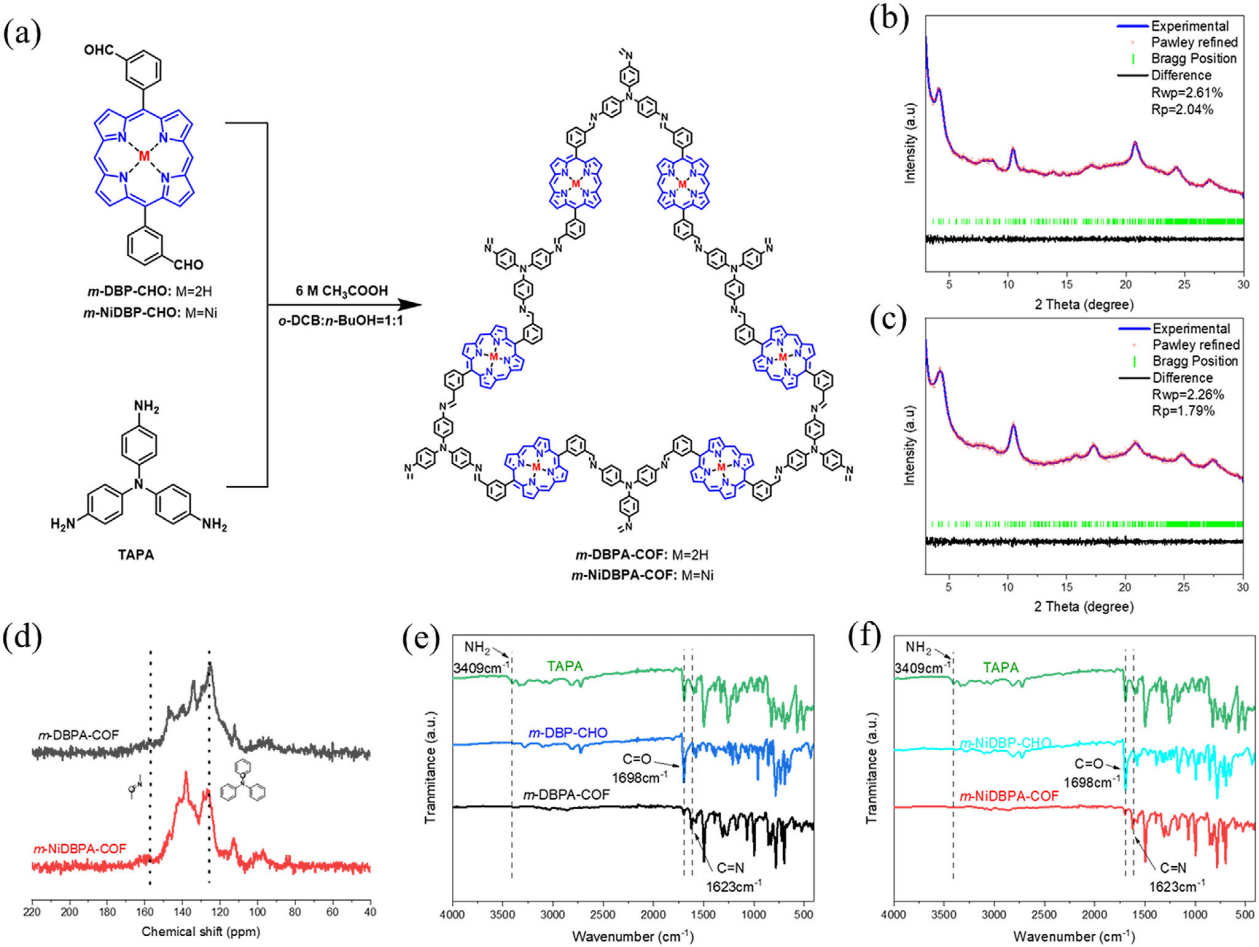
a) Design and synthesis of *m*‐DBPA‐COF and *m*‐NiDBPA‐COF. Experimental and AA stacking‐simulated PXRD patterns of b) *m*‐DBPA‐COF and c) *m*‐NiDBPA‐COF. d) Solid‐state ^13^C NMR spectra of *m*‐DBPA‐COF and *m*‐NiDBPA‐COF. e) FT‐IR spectra of *m*‐DBPA‐COF and monomers. f) FT‐IR spectra of *m*‐NiDBPA‐COF and monomers.

As shown in **Figure** **1**a, *m*‐DBPA‐COF and *m*‐NiDBPA‐COF were synthesized via the condensation reaction between TAPA and the corresponding aldehyde‐based porphyrin monomers. The powder X‐ray diffraction (PXRD) experiment was conducted to elucidate the crystallinity of *m*‐DBPA‐COF and *m*‐NiDBPA‐COF. As illustrated in Figure 1b,c, both materials exhibit a primary PXRD peak at a 2θ value of 4.2 °, corresponding to the (100) reflection. The Pawley refinement was performed (Figure 1b,c) to optimize the crystal structures of the materials. Notably, the calculated diffraction patterns based on the AA eclipsed stacking model exhibited excellent agreement with the experimental PXRD patterns, indicating that both *m*‐DBPA‐COF and *m*‐NiDBPA‐COF adopted an AA layer stacking structure.

In Figure 1d, the solid‐state ^13^C nuclear magnetic resonance (NMR) spectra indicated that both *m*‐DBPA‐COF and *m*‐NiDBPA‐COF exhibited a characteristic peak at 158 ppm, attributable to the C═N bond.^[^ ^35^ ^]^ In addition, a typical peak at 125 ppm could be observed, corresponding to the triphenylamine carbon atoms,^[^ ^46^ ^]^ and other broad peaks spanning 110–150 ppm were ascribed to the carbon atoms within the porphyrin units, consistent with previously reported porphyrin polymers.^[^ ^47^ ^]^ Moreover, as shown in Fourier‐transform infrared (FT‐IR) spectra (Figure 1e,f), the near disappearance of the C═O and NH_2_ stretching bands typically observed in the monomers, alongside the emergence of a prominent absorption signal at 1623 cm^−1^ attributable to the C═N bond,^[^ ^41^ ^]^ confirmed the successful construction of *m*‐DBPA‐COF and *m*‐NiDBPA‐COF.

**Figure 2:**
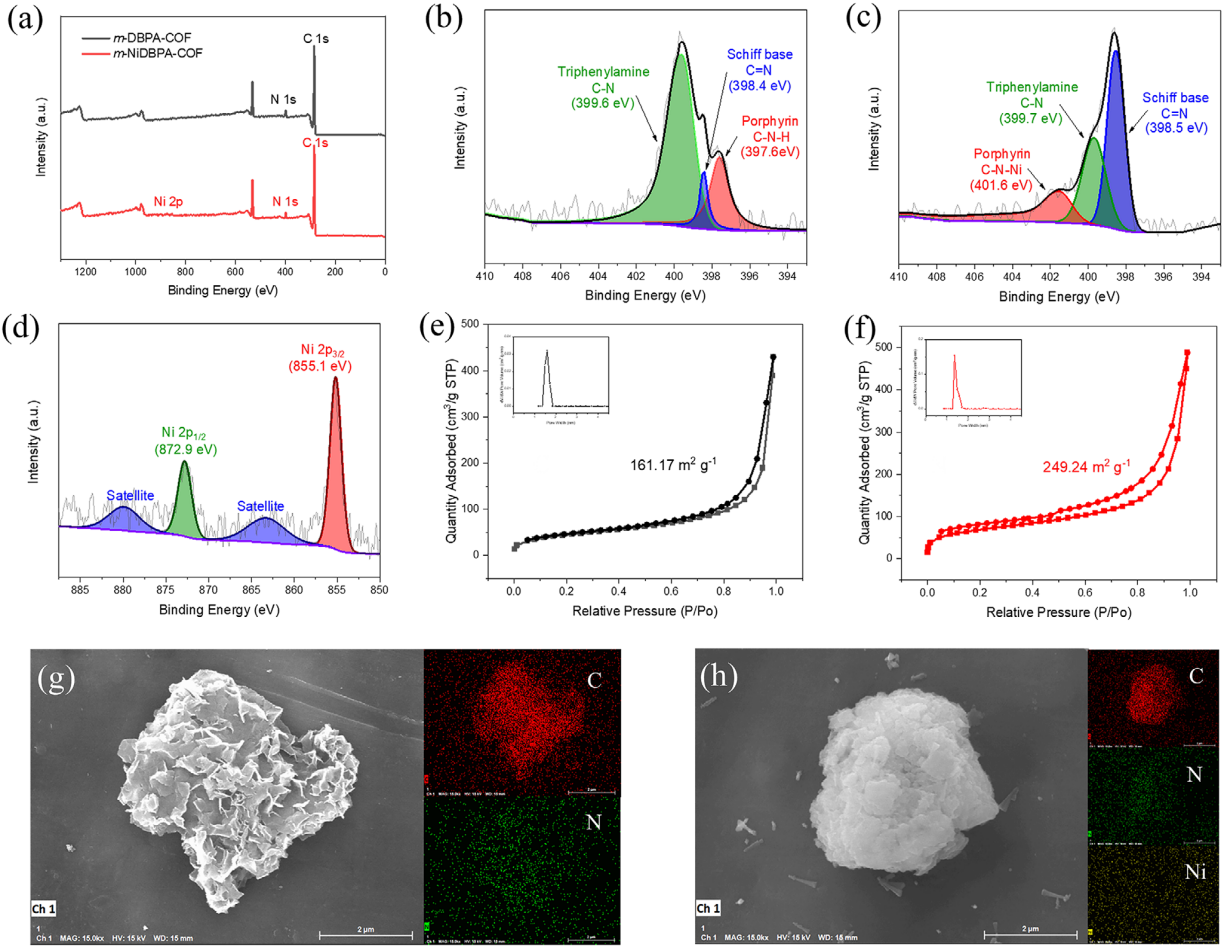
a) XPS survey scan spectra of *m*‐DBPA‐COF and *m*‐NiDBPA‐COF. b) High resolution N 1s XPS spectrum of *m*‐DBPA‐COF. c) High resolution N 1s XPS spectrum of *m*‐NiDBPA‐COF. d) High resolution Ni 2p XPS spectrum of *m*‐NiDBPA‐COF. N_2_ sorption isotherm and pore size distribution of e) *m*‐DBPA‐COF and f) *m*‐NiDBPA‐COF. SEM images and EDS elemental mapping of g) *m*‐DBPA‐COF and h) *m*‐NiDBPA‐COF.

To further confirm the successful synthesis of *m*‐DBPA‐COF and *m*‐NiDBPA‐COF, X‐ray photoelectron spectroscopy (XPS) measurements were performed (**Figure** **2**a–d). As shown in Figure 2b, the peaks at 399.6, 398.4, and 397.6 eV in the high‐resolution N 1s spectrum corresponded to C‐N from the triphenylamine structure, imine C═N, and pyrrolic N from the porphyrin structure in the *m*‐DBPA‐COF. In contrast, for *m*‐NiDBPA‐COF, the pyrrolic N peaks exhibited a positive shift compared with *m*‐DBPA‐COF (Figure 2c), implying that the introduction of Ni ions into the porphyrin ring decreased its electron density. This shift might be attributed to LMCT from the porphyrin ligand to Ni ions.^[^ ^48^ ^]^ Furthermore, high‐resolution XPS Ni 2p spectrum of *m*‐NiDBPA‐COF was acquired to elucidate the electronic states of the embedded Ni ions. As shown in Figure 2d, the peaks at binding energies of 872.9 and 855.1 eV were attributed to Ni(II).^[^ ^49^, ^50^ ^]^

The N_2_ adsorption/desorption isotherms were performed to assess the pore properties of *m*‐DBPA‐COF and *m*‐NiDBPA‐COF (Figure 2e,f). Both materials exhibited type‐I isotherms, confirming their microporous nature. The Brunauer‐Emmett‐Teller (BET) surface areas of *m*‐DBPA‐COF and *m*‐NiDBPA‐COF were determined to be 161.17 and 249.24 m^2^ g^−1^, respectively. Additionally, the predominant pore sizes for both materials were determined to be approximately 1.5 nm, thereby confirming that the incorporation of Ni ions did not alter the stacking mode of the material. The surface morphologies of *m*‐DBPA‐COF and *m*‐NiDBPA‐COF were assessed using scanning electron microscopy (SEM). The SEM images revealed that both materials exhibit a plate‐like particulate morphology (Figure 2g,h). Energy‐dispersive spectroscopy (EDS) elemental mapping (Figure 2g,h) revealed that carbon and nitrogen were uniformly distributed across both materials, and nickel was uniformly present in *m*‐NiDBPA‐COF, consistent with the XPS results.

### 2.2 Photoelectronic Properties of *m*‐DBPA‐COF and *m*‐NiDBPA‐COF

**Figure 3:**
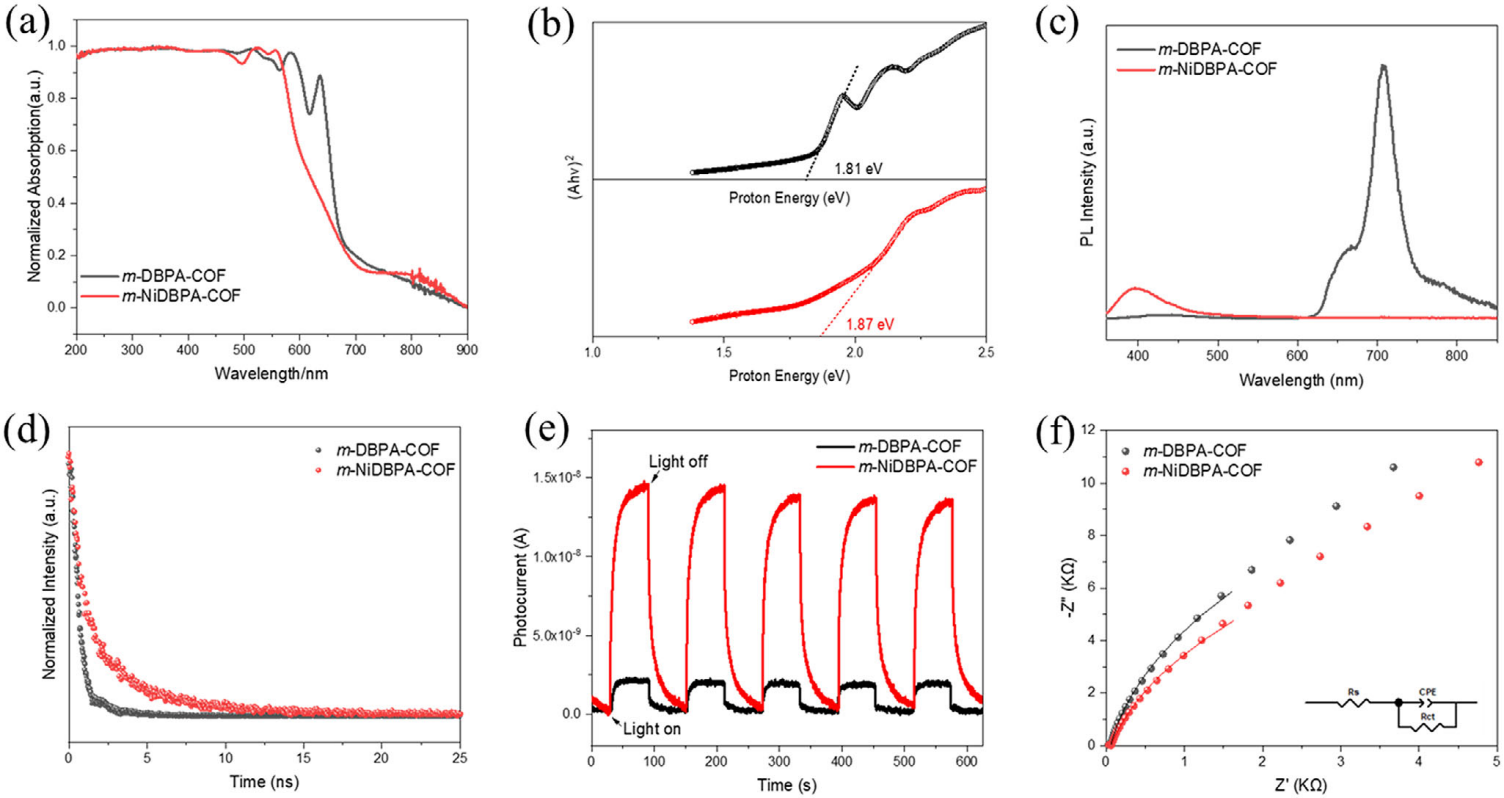
a) UV–vis diffuse reflection absorption spectra of *m*‐DBPA‐COF and *m*‐NiDBPA‐COF. b) Tauc plot and calculated bandgap of *m*‐DBPA‐COF and *m*‐NiDBPA‐COF. c) Photoluminescence spectra of *m*‐DBPA‐COF and *m*‐NiDBPA‐COF at an excitation wavelength of 340 nm. d)Time‐resolved photoluminescence spectra of *m*‐DBPA‐COF and *m*‐NiDBPA‐COF at an excitation wavelength of 375 nm. e) Transient photocurrent responses of *m*‐DBPA‐COF and *m*‐NiDBPA‐COF. f) Electrochemical impedance spectra (EIS) of *m*‐DBPA‐COF and *m*‐NiDBPA‐COF.

As shown in the solid‐state ultraviolet–visible (UV–vis) diffuse reflectance spectra (**Figure** **3**a), *m*‐DBPA‐COF initially exhibited four characteristic Q‐band peaks. However, following the incorporation of Ni ions, these four peaks converged into two, consistent with the typical spectral behavior of metal porphyrins.^[^ ^51^ ^]^ Moreover, *m*‐NiDBPA‐COF exhibited a blue shift in light absorption compared to *m*‐DBPA‐COF, a phenomenon likely attributable to LMCT from the porphyrin ligand to Ni ions.^[^ ^52^ ^]^ Additional band gap calculations were conducted using Kubelka‐Munk conversion reflectance spectroscopy. As shown in Figure 3b, the optical band gaps of *m*‐DBPA‐COF and *m*‐NiDBPA‐COF were determined to be 1.81 and 1.87 eV, respectively. These results indicated that the incorporation of Ni ions led to an expansion of the optical band gaps.

To further investigate the photogenerated charge transfer and separation properties of *m*‐DBPA‐COF and *m*‐NiDBPA‐COF, photoluminescence (PL) emission spectra were examined (Figure 3c). Compared with *m*‐DBPA‐COF, the quenched PL signal indicated a more efficient separation of photoinduced electron‐hole pairs in *m*‐NiDBPA‐COF, which might be attributed to the LMCT effect that accelerated electron migration from the porphyrin ligand to Ni ions. To estimate the excited‐state lifetimes of *m*‐DBPA‐COF and *m*‐NiDBPA‐COF in the solid state, time‐resolved PL decay measurements were performed (Figure 3d). Analysis of the decay curves via reconvolution fitting confirmed that a double exponential decay model was appropriate in each case (Figure S18 and Table S2, Supporting Information). Notably, *m*‐NiDBPA‐COF exhibited an extended carrier lifetime, which was attributed to a substantial reduction in photoexcited electron‐hole pair recombination, resulting from rapid charge transfer from the porphyrin ring to the coordinated Ni ions.^[^ ^53^ ^]^

Transient photocurrent response measurements and electrochemical impedance spectra for *m*‐DBPA‐COF and *m*‐NiDBPA‐COF were subsequently performed (Figure 3e,f; Table S3, Supporting Information). Under light illumination, *m*‐NiDBPA‐COF exhibited lower charge transfer resistance and higher photocurrent intensity compared to *m*‐DBPA‐COF. These results, consistent with the observed emission decay profiles, further confirmed the superior charge carrier transport efficiency of *m*‐NiDBPA‐COF. Collectively, these tests demonstrated that the incorporation of Ni ions significantly enhanced charge separation and transport efficiency, thereby substantially improving the photochemical properties of *m*‐NiDBPA‐COF.

### 2.3 Photocatalytic Performance of *m*‐NiDBPA‐COF for CO_2_ Cycloaddition to Aziridines

Motivated by its pronounced and broad visible‐light absorption and exceptional charge carrier transport efficiency, *m*‐NiDBPA‐COF was evaluated for its photocatalytic performance in the CO_2_ cycloaddition to aziridines.

**Figure 4:**
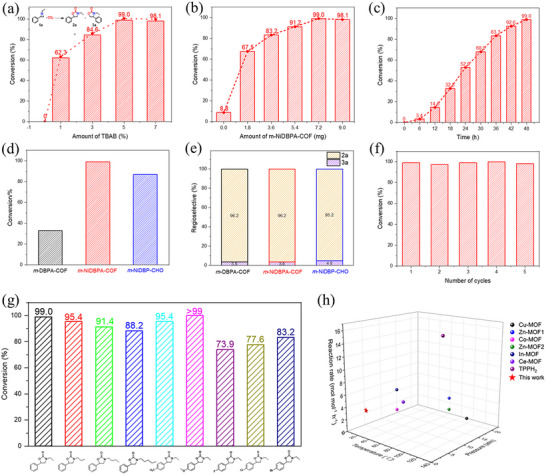
a) Effect of TBAB amount. b) Effect of *m*‐NiDBPA‐COF loading. c) Effect of reaction time. d) Catalytic performance comparison and e) regioselectivity for CO_2_ cycloaddition reaction with different catalysts; Reaction conditions: 1‐ethyl‐2‐phenylaziridine (2 mmol), catalyst (0.5 mol% porphyrin centers/metal ions), TBAB (32.2 mg, 5 mol%), 1 atm CO_2_, 300 W Xenon lamp, reaction time to 48 h. f) Recyclability of *m*‐NiDBPA‐COF. g) Catalytic performance of *m*‐NiDBPA‐COF over various aziridines. h) Comparison of catalytic CO_2_ cycloaddition activities for typical heterogeneous catalysts. The conversion and regioselectivity were determined by ^1^H NMR.

To investigate the photocatalytic performance of *m*‐NiDBPA‐COF, we employed the cycloaddition reaction between CO_2_ and 1‐ethyl‐2‐phenylaziridine as a model system. The effects of reaction conditions, including the tetrabutylammonium bromide (TBAB) cocatalyst amount, catalyst loading and reaction time, were evaluated. The conversion of 1‐ethyl‐2‐phenylaziridine increased from 0% to 99.0% as the TBAB concentration rose from 0 to 5 mol% (**Figure** **4**a). However, a slight decline in conversion was observed when the TBAB concentration further increased from 5 to 7 mol%. Consequently, 5 mol% TBAB was selected for subsequent investigations. Figure 4b illustrates the correlation between catalyst loading and the conversion of 1‐ethyl‐2‐phenylaziridine. The introduction of additional catalytic sites (Ni ions) substantially enhanced the conversion efficiency. Notably, increasing the catalyst amount from 0 mg to 7.2 mg (0.5 mol% Ni ions) resulted in a conversion rate of 99.0%. As shown in Figure 4c, the reaction time significantly affected the conversion of 1‐ethyl‐2‐phenylaziridine, with a conversion rate of 99.0% achieved after 48 h. Therefore, 5 mol% TBAB cocatalyst and catalysts loaded with 0.5 mol% Ni ions were adopted for the CO_2_ cycloaddition reaction for 48 h.

As shown in Figure 4d, under identical conditions, the *m*‐NiDBPA‐COF exhibited superior photocatalytic performance compared to the metal‐free *m*‐DBPA‐COF, suggesting that the incorporation of Ni ions in the porphyrin cores enhanced CO_2_ cycloaddition catalysis. Furthermore, in comparison with the monomer *m*‐NiDBP‐CHO, the *m*‐NiDBPA‐COF demonstrated improved catalytic efficiency, achieving a 99.0% conversion of 1‐ethyl‐2‐phenylaziridine. This enhancement was likely attributable to its effective CO_2_ adsorption and the efficient transfer of photogenerated charges. Moreover, high regioselectivity was observed for all catalysts (Figure 4e). The exceptional recyclability of *m*‐NiDBPA‐COF was evidenced by only a minor reduction in catalytic activity observed after five cycles (Figure 4f). Additionally, PXRD and FT‐IR analyses of the recovered *m*‐NiDBPA‐COF (Figure S23, Supporting Information) confirmed that its chemical composition and porous structure were well preserved. Furthermore, a diverse set of aziridines was employed to systematically examine the substrate scope in the *m*‐NiDBPA‐COF‐catalyzed CO_2_ cycloaddition reaction. As illustrated in Figure 4g, high conversion rates were achieved for the majority of investigated aziridines, indicating that *m*‐NiDBPA‐COF exhibited excellent substrate tolerance. However, with increasing alkyl chain length, the catalytic performance of *m*‐NiDBPA‐COF for aziridines bearing bulkier chains was diminished relative to that observed for smaller aziridines, thereby demonstrating its size selectivity. Additionally, the presence of an electron‐withdrawing fluorine, chlorine, or bromine substituent in aziridines resulted in a slight decrease in conversion.

Encouragingly, compared with previous thermal catalysts, the *m*‐NiDBPA‐COF catalyzed CO_2_ cycloaddition with aziridines occurred with the conversion of aziridines over 99% under significantly mild catalytic conditions (1 atm CO_2_ with no heating required) as shown in Figure 4h and Table S7 (Supporting Information). Even more interestingly, the photocatalytic reaction rate using *m*‐NiDBPA‐COF with less metal ion centers involved was comparable to those with most thermal catalysts (Figure 4h; Table S7, Supporting Information). To elucidate the origin of the photocatalytic activity observed in *m*‐NiDBPA‐COF, additional control experiments were conducted. As demonstrated in **Table** **1**, the absence of co‐catalyst TBAB resulted in a dramatic reduction in the conversion of 1‐ethyl‐2‐phenylaziridine to negligible levels (entry 2). Moreover, in the absence of *m*‐NiDBPA‐COF, the conversion of 1‐ethyl‐2‐phenylaziridine was exceedingly low (entry 3), thereby confirming the critical role of *m*‐NiDBPA‐COF in facilitating photocatalysis. Under dark conditions (entry 4), the conversion of 1‐ethyl‐2‐phenylaziridine sharply decreased to 11.4%. Additionally, a slight reduction in conversion was observed at 25 °C (entry 5). These results indicated that light was essential for this reaction when catalyzed by *m*‐NiDBPA‐COF, suggesting that the process was predominantly photo‐driven. Meanwhile, sodium persulfate and sodium ascorbate were added, respectively, to conduct the electron (e^−^) and hole (h^+^) trapping tests (entries 6 and 7). In the presence of sodium ascorbate (h^+^ sacrificial agent) and sodium persulfate (e^−^ sacrificial agent), the conversion of 1‑ethyl‑2‑phenylaziridine decreased to 48.1% and 79.8%, respectively, indicating that both photogenerated electrons and holes contributed to the photocatalytic process.

**Table 1:** Cycloaddition reaction between CO_2_ and 1‐ethyl‐2‐phenylaziridine under different conditions.^a)^

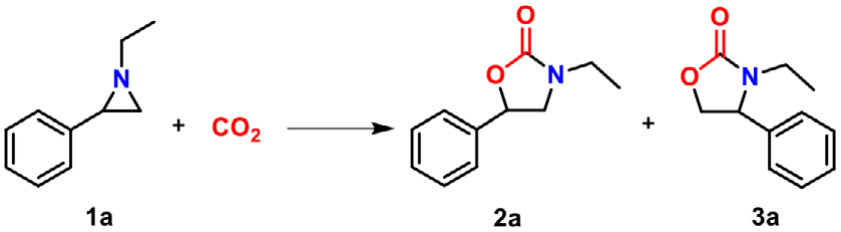					
Entry	Catalyst	Cocatalyst	Reaction Condition	Conversion/%^b)^	Regio‐sel^b)^, ^c)^
1	*m*‐NiDBPA‐COF	TBAB	300 W Xenon lamp	99.0	96.2: 3.8
2^d)^	*m*‐NiDBPA‐COF	‐	300 W Xenon lamp	0	‐
3^e)^	‐	TBAB	300 W Xenon lamp	7.2	95.2: 4.8
4^f)^	*m*‐NiDBPA‐COF	TBAB	Dark	11.4	95.2: 4.8
5^g)^	*m*‐NiDBPA‐COF	TBAB	300 W Xenon lamp	93.6	97.1: 2.9
6^h)^	*m*‐NiDBPA‐COF	TBAB	300 W Xenon lamp, in the presence of e^−^ SA	79.8	97.1: 2.9
7^i)^	*m*‐NiDBPA‐COF	TBAB	300 W Xenon lamp, in the presence of h^+^ SA	48.1	97.1: 2.9

^a)^ Reaction conditions: 1‐ethyl‐2‐phenylaziridine (2 mmol), *m*‐NiDBPA‐COF (7.2 mg, 0.5 mol% Ni ions), TBAB (32.2 mg, 5 mol%), 1 atm CO_2_, 300 W Xenon lamp, reaction time to 48 h;

^b)^ The conversion and regioselectivity were determined by ^1^H NMR;

^c)^ Molar ratio of 2a to 3a;

^d)^ In the absence of the cocatalyst TBAB;

^e)^ In the absence of the catalyst;

^f)^ Under dark conditions at 30 °C;

^g)^ An EtOH circulating system maintained the reaction temperature at 25 °C;

^h)^ In the presence of 19.8 mg sodium persulfate as e^−^ sacrificial agent;

^i)^ In the presence of 23.8 mg sodium ascorbate as h^+^ sacrificial agent.

### 2.4 Mechanism for Photocatalytic CO_2_ Cycloaddition to Aziridines

**Figure 5:**
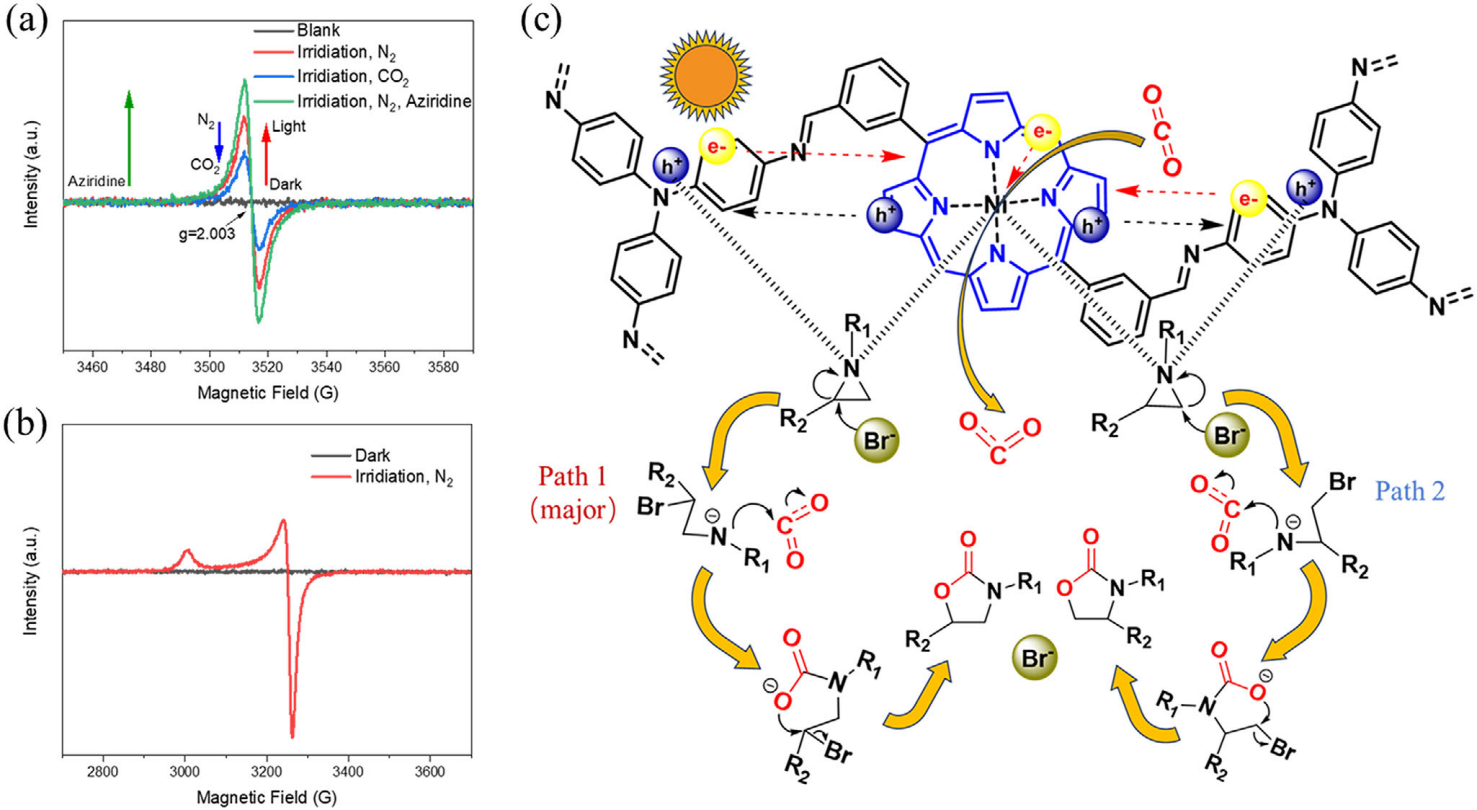
a) EPR spectra of *m*‐NiDBPA‐COF under different conditions (TEMPO as a free electron capture agent). b) EPR spectra of *m*‐NiDBPA‐COF in the dark and under light irradiation. c) Proposed photocatalytic CO_2_ cycloaddition reaction mechanism.

To investigate the photocatalytic and charge transfer behavior of *m*‐NiDBPA‐COF, in situ electron paramagnetic resonance (EPR) measurements were performed. As shown in **Figure** **5**a, irradiation of *m*‐NiDBPA‐COF under N_2_ atmosphere resulted in an EPR signal with a g‐value of 2.003, which was attributed to free electrons.^[^ ^54^, ^55^ ^]^ The signal intensity decreased significantly when CO_2_ replaced N_2_, suggesting rapid electron transfer to CO_2_ that facilitated its activation. In contrast, the addition of aziridine (1‐ethyl‐2‐phenylaziridine) to the system markedly increased the electron signal intensity, indicating an enhancement in electron generation.^[^ ^48^, ^56^ ^]^ A similar result was observed for the nickel species (Figure 5b). Under light irradiation, new EPR signals were detected, which were attributed to Ni(I) species coordinated by porphyrin ligands.^[^ ^57^ ^]^ This phenomenon was ascribed to the conversion of low‐spin Ni(II) to high‐spin Ni(I) via a LMCT process.

Based on experimental findings and previous reports,^[^ ^14^, ^30^, ^58^ ^]^ the following mechanism for the photocatalytic CO_2_ cycloaddition reaction over *m*‐NiDBPA‐COF is proposed (Figure 5c). Initially, under light irradiation, *m*‐NiDBPA‐COF functions as a light harvester, generating electron‐hole pairs. The photogenerated electrons are rapidly transferred to Ni(II), leading to the prompt partial reduction of Ni(II) to Ni(I), while the photogenerated holes migrate to the triphenylamine units of *m*‐NiDBPA‐COF. Then, CO_2_ is activated by abundant photogenerated electrons and Ni(I) species, whereas Ni(II) species and photogenerated holes, acting as Lewis acidic sites,^[^ ^59^ ^]^ facilitate the activation of the aziridine substrate. The ring‐opening of aziridines is promoted by Br anions through two distinct pathways, as represented by path 1 (the major path, with more stable carbamate salt intermediates) or path 2. Subsequently, the activated CO_2_ is inserted into the ring‐opened aziridine intermediate, and the reaction culminates in an intramolecular ring‐closure to yield oxazolidinone products, concurrently regenerating the catalyst and releasing bromide anions.

## 3 Conclusion

In summary, two novel *trans*‐A_2_B_2_‐type porphyrin‐based D‐A type COFs for photocatalytic CO_2_ cycloaddition to aziridines have been successfully designed and synthesized. Experimental analyses reveal that the incorporation of nickel porphyrins markedly enhances the transfer and separation of photoinduced charge carriers through ligand‐to‐metal charge transfer (LMCT) and the electron push‐pull effect characteristic inherent in D‐A configurations. Moreover, the incorporated Ni ions provide Lewis acidic sites that facilitate substrate interactions. Under visible light‐assisted and mild conditions, *m*‐NiDBPA‐COF effectively catalyzes the cycloaddition of CO_2_ with aziridines. Notably, after five consecutive cycles, no significant reduction in catalytic activity is observed, and the catalyst demonstrates broad applicability across various aziridine substrates. To the best of our knowledge, metalloporphyrin‐based COF photocatalysts was unprecedentedly applied for the CO_2_ cycloaddition to aziridines with no elevated pressure and heating required. This work not only presents a design strategy for efficient heterogeneous porphyrin‐based catalysts, but also broadens the scope of COFs in photocatalysis.

## Conflict of Interest

The authors declare no conflict of interest.

## Supporting information



Supporting Information

## Data Availability

The data that support the findings of this study are available in the supplementary material of this article.
