# Supplementary material for: Trans‐A2B2‐Type Metalloporphyrin‐Based Donor–Acceptor Covalent Organic Frameworks for Efficient Photocatalytic CO2 Cycloaddition to Aziridines
Source: Adv Sci (Weinh). 2025 Nov 12;13(6):e13754. doi: 10.1002/advs.202513754 (PMC12866726; doi:10.1002/advs.202513754)
Supplement: Supplementary file 1 — Supporting Information [file ADVS-13-e13754-s001.docx]

Supporting Information

***Trans*-A_2_B_2_-type Metalloporphyrin-based Donor-acceptor Covalent Organic Frameworks for Efficient Photocatalytic CO_2_ Cycloaddition to Aziridines**

*Ji Xiong, Minghui Chen, Yunhao Xu, Yaqing Feng, Jian Song, and Bao Zhang**

**Contents**

[1. Experimental and characterization details S2](#_Toc211787272)

[**1.1 Chemicals and reagents S2**](#_Toc211787273)

[**1.2 Characterization methods S2**](#_Toc211787274)

[2. Synthetic Procedures S3](#_Toc211787275)

[3. Preparation of aziridines S11](#_Toc211787276)

[4. Catalytic CO_2_ cycloaddition reaction S16](#_Toc211787277)

[5. Characterizations S17](#_Toc211787278)

[**5.1 Structure characterization S17**](#_Toc211787279)

[**5.2 Catalytic performance of *m*-NiDBPA-COF for CO_2_ cycloaddition to aziridines S18**](#_Toc211787280)

[6. References S31](#_Toc211787281)

**1.** **Experimental and characterization details**

**1.1 Chemicals and reagents**

All chemicals and reagents were used as received from commercial sources without further purification. 1,3-phthalaldehyde, neopentyl glycol, *N, N*-dimethylformamide (DMF), tetrahydrofuran (THF), 1,2-dichlorobenzene (*o*-DCB), *n*-butanol (*n*-BuOH), tris(4-aminophenyl)amine (TAPA), Nafion aqueous solution, tetrabutylammonium bromide (TBAB), tetrabutylammonium chloride (TBAC) and tetrabutylammonium iodide (TBAI) were purchased from Aladdin industrial corporation, Shanghai, China. Pyrrole, *p*-toluenesulfonic acid monohydrate, trifluoroacetic acid, triethylamine and *p*-chloranil were purchased from Kmart chemical technology limited company, Tianjin, China. Nickel(II) acetate tetrahydrate (Ni(OAc)_2_•4H_2_O) and chloroform-d (CDCl_3_) were purchased from Shanghai Bide Pharmaceutical Technology Co., Ltd. Benzene, dichloromethane (DCM), petroleum ether (PE), ethyl acetate (EA), ethanol (EtOH), acetonitrile (CH_3_CN), Liquid bromine (Br_2_), hydrochloric acid, sodium sulfate (Na_2_SO_4_), sodium bicarbonate (NaHCO_3_), and silicone (200-300 mesh) were purchased from Jiangtian chemical technology limited company, Tianjin, China.

**1.2 Characterization methods**

Liquid 1D ^1^H nuclear magnetic resonance (NMR) spectra and ^13^C NMR of all monomers were recorded on a Bruker AVANCE IIITM HD 400 MHz NanoBAY. 600 MHz solid-state ^13^C NMR spectra were measured on the JEOL JNM ECZ600R. The HR-MS (ESI) data were recorded with a MICRO–Q TOF mass spectrometer by using the ESI technique at 10 eV. Fourier-transform infrared (FT-IR) spectra were recorded on Nicolet6700 instrument (400-4000 cm^-1^ region). Powder X ray diffraction (PXRD) data were collected on Rigaku Smartlab8KW using Cu Kα radiation at 40 kV,40 mA power. X-ray photoelectron spectroscopy (XPS) measurements were carried out on ThermoFisher Scientific K-Alpha+ using monochromated Al Kα radiation as the excitation source and C 1s peak at 284.8 eV as internal standard. Brunauer-Emmett-Teller (BET) surface areas were measured by nitrogen adsorption and desorption at 77 K using Autosorb-iQ2-MP and the samples were activated at 100 °C for 8 h under vacuum before analysis. Pore size distributions and pore volumes were derived from the adsorption branches of the isotherms using the density functional theory (DFT) pore mode. Field Emission Scanning Electron Microscopy (SEM) and energy dispersive spectroscopy (EDS) were measured on Hitachi S-4800. UV-Visible diffuse reflectance spectra (DRS) were obtained by Lambda 750, Perkin Elmer with an integrating sphere mode. The steady photoluminescence (PL) data and fluorescence quantum yield were measured by FLS1000 fluorescence spectrometer in the solid state. Electron Paramagnetic Resonance (EPR) Spectra was measured by Bruker EMXnano. Electrochemical properties of the material frameworks were measured through a three-electrode system in an electrochemical workstation with a brand of CHI66, Chenhua. For these measurements, 5 mg COFs was dispersed into the mixed solvent of 2 mL EtOH and 50 μL 5wt% Nafion aqueous solution by ultrasonic 60 min. After that, 30 μL suspension was deposited onto clean Φ3 GCE as the working electrode. And the Ag/AgCl electrode worked as the reference electrode and Pt flake was acted as the counter electrode. 0.1 M Na_2_SO_4_ aqueous solution acted as electrolyte during the measurements.

**2. Synthetic Procedures**

**Scheme S1**. Synthesis of dipyrromethane

**Synthesis of dipyrromethane**

Dipyrrylmethane was synthesized referred to the literature procedure [1]. Distilled pyrrole (30 mL, 430 mmol) was added into 300 mL 0.1 M HCl solution in ice bath in the dark. Subsequently, formaldehyde solution (6 mL, 81.3 mmol) was added into the solution dropwise and further reacted with vigorous stirring for 40 min. Then, the resulting suspension was neutralized by saturated Na_2_CO_3_ aqueous solution and extracted by DCM. The organic layer was dried by Na_2_SO_4_ and concentrated under reduced pressure, and the unreacted pyrrole was distilled off under vacuum to yield crude product. The crude product was purified by column chromatography (PE/DCM/EA (16:3:1, by vol.), silica gel, 200-300 mesh) to afford the white crystal product. Yield: 6.80 g (57.2%).

**Scheme S2**. Synthesis of 4-(5,5-dimethyl-1,3-dioxan-2-yl)benzaldehyde

**Synthesis of** **3-(5,5-dimethyl-1,3-dioxan-2-yl)benzaldehyde**

A solution of 1,3-phthalaldehyde (6.7 g, 50.0 mmol), neopentyl glycol (5.2 g, 50.0 mmol) and *p*-toluenesulfonic acid monohydrate (1.9 g, 10.0 mmol) in benzene (120 mL) was heated at 85 ºC for 5 h. After cooling down to room temperature, benzene was evaporated and the residue was diluted with DCM. The solution was washed with water and brine. The organic layer was separated, dried over anhydrous Na_2_SO_4,_ filtered and concentrated in vacuo to 1/4 by volume. The concentrated solution was then purified by column chromatography (EA/hexane (1:10, by vol.), silica gel, 200-300 mesh) to give a white solid. Yield: 8.0 g (72.7%).  ^1^H NMR (400 MHz, CDCl_3_) *δ* 10.02 (s, 1H), 8.03 (s, 1H), 7.86 (d, *J* = 7.6 Hz, 1H), 7.77 (d, *J* = 7.7 Hz, 1H), 7.53 (t, *J* = 7.6 Hz, 1H), 5.45 (s, 1H), 3.78 (d, *J* = 11.1 Hz, 2H), 3.67 (d, *J* = 10.9 Hz, 2H), 1.29 (s, 4H), 0.80 (s, 3H). ^13^C NMR (400 MHz, CDCl_3_) *δ* 192.13, 139.69, 136.43, 132.30, 129.74, 129.04, 127.98, 100.69, 77.67, 30.27, 23.06, 21.86. HR-MS (ESI): m/z calculated for C_13_H_16_O_3_ 220.1009, found 221.1172 [M+H]^+^.


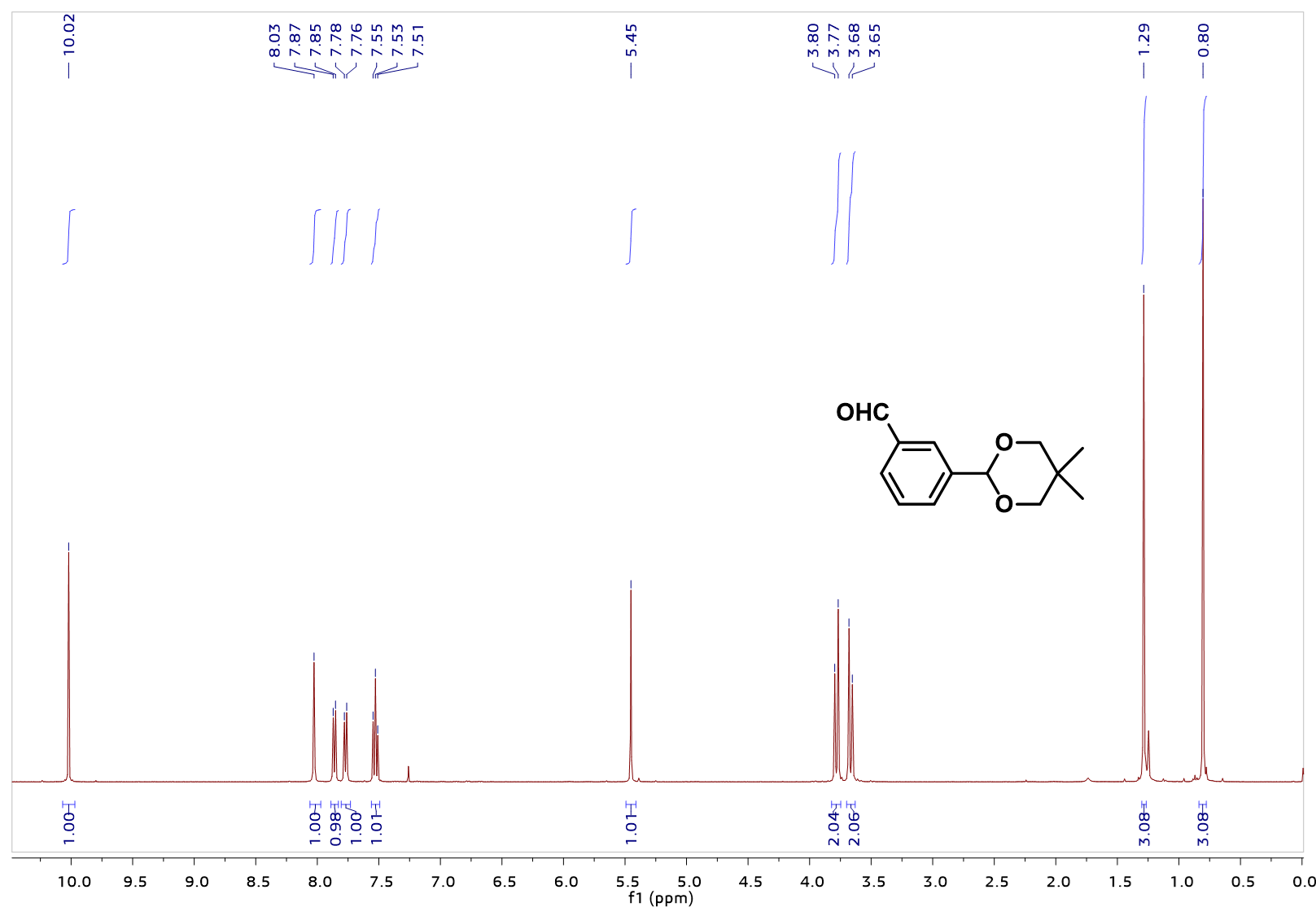


**Figure S1**. ^1^H NMR spectrum of 4-(5,5-dimethyl-1,3-dioxan-2-yl)benzaldehyde


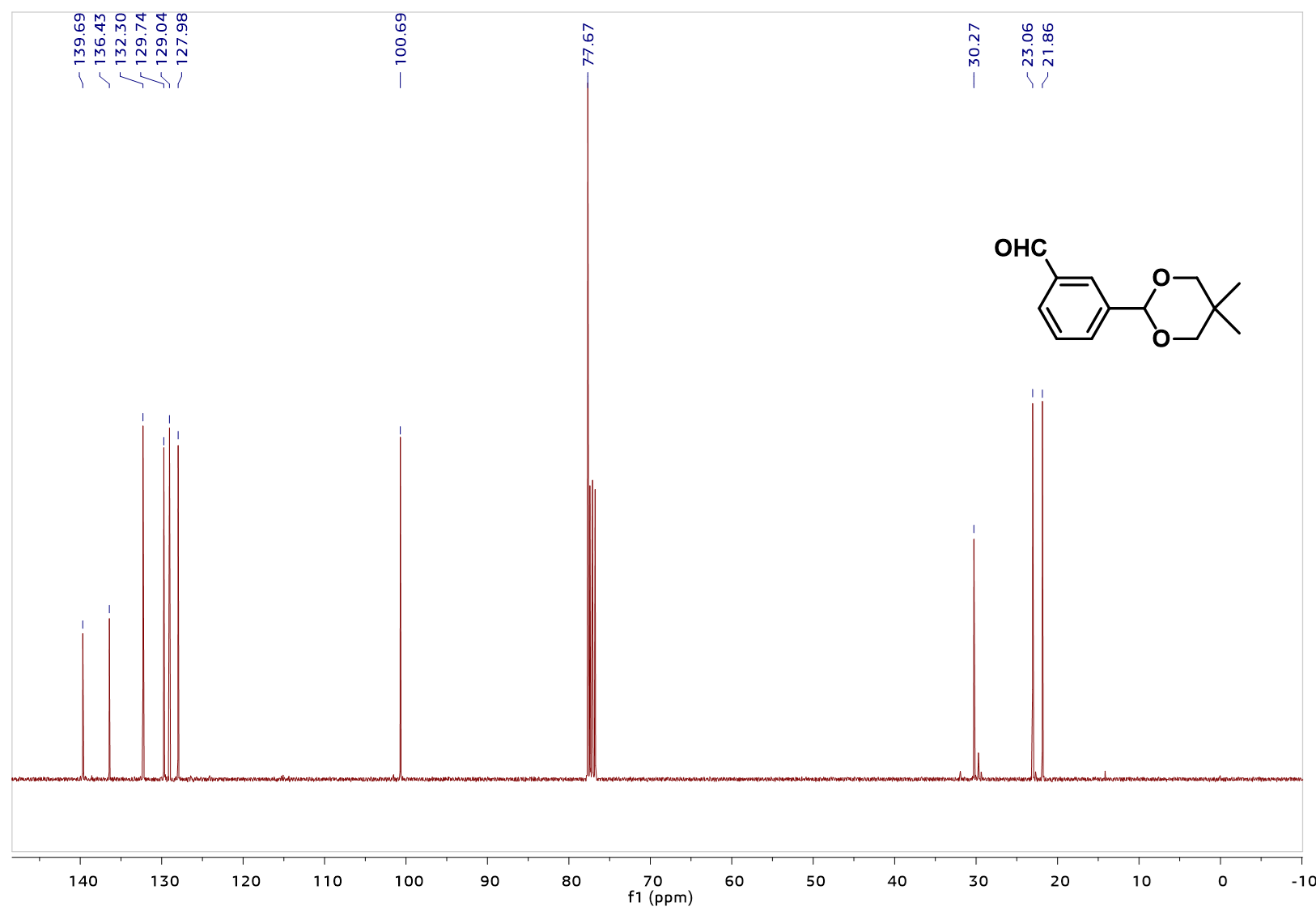


**Figure S2**. ^13^C NMR spectrum of 4-(5,5-dimethyl-1,3-dioxan-2-yl)benzaldehyde

**Scheme S3**. Synthesis of [5,15-di(3-benzaldehyde)porphyrinato]nickel(II)

**Synthesis of** **5,15-di((3-(1,3-dioxa-5,5-dimethylcyclohex-2-yl)phenyl)porphyrin**

A solution of dipyrrylmethane (1.4 g, 10.0 mmol) and 3-(5,5-dimethyl-1,3-dioxan-2-yl)benzaldehyde (2.2 g, 10.0 mmol) in dry DCM (500 mL) was purged with argon for 30 min. To this solution trifluoroacetic acid (0.37 mL, 5.00 mmol) was added. The reaction mixture (protected from light) was stirred at room temperature for 4 h under argon atmosphere. To this resulting black color solution, 2,3-dichloro-5,6-dicyano-1,4-benzoquinone (3.4 g, 15.0 mmol) was added in one portion and stirred for additional 1 h. Triethylamine was added to neutralize the reaction mixture. The resulting reaction mixture was concentrated in vacuo to 1/4 by volume and then purified by column chromatography (EA/DCM (50:1), silica gel, 200-300 mesh) to give a purple solid. Yield: 925.0 mg (26.8%). ^1^H NMR (400 MHz, CDCl_3_) *δ* 10.32 (s, 2H), 9.39 (d, *J* = 4.6 Hz, 4H), 9.08 (d, *J* = 4.5 Hz, 4H), 8.40 (s, 2H), 8.26 (d, *J* = 6.7 Hz, 2H), 8.01 (d, *J* = 7.9 Hz, 2H), 7.84 (t, *J* = 7.6 Hz, 2H), 5.72 (s, 2H), 3.88 (d, *J* = 11.2 Hz, 4H), 3.77 (d, *J* = 11.1 Hz, 4H), 1.37 (s, 6H), 0.84 (s, 6H). -3.14 (s, 2H). ^13^C NMR (400 MHz, CDCl_3_) *δ* 147.16, 145.22, 141.30, 137.36, 135.35, 132.75, 131.63, 131.19, 127.08, 125.58, 118.79, 105.27, 102.07, 77.87, 30.36, 23.15, 21.93. HR-MS (MALDI-TOF): m/z calculated for C_44_H_42_N_4_O_4_ 690.3206, found 691.3283 [M]^+^.


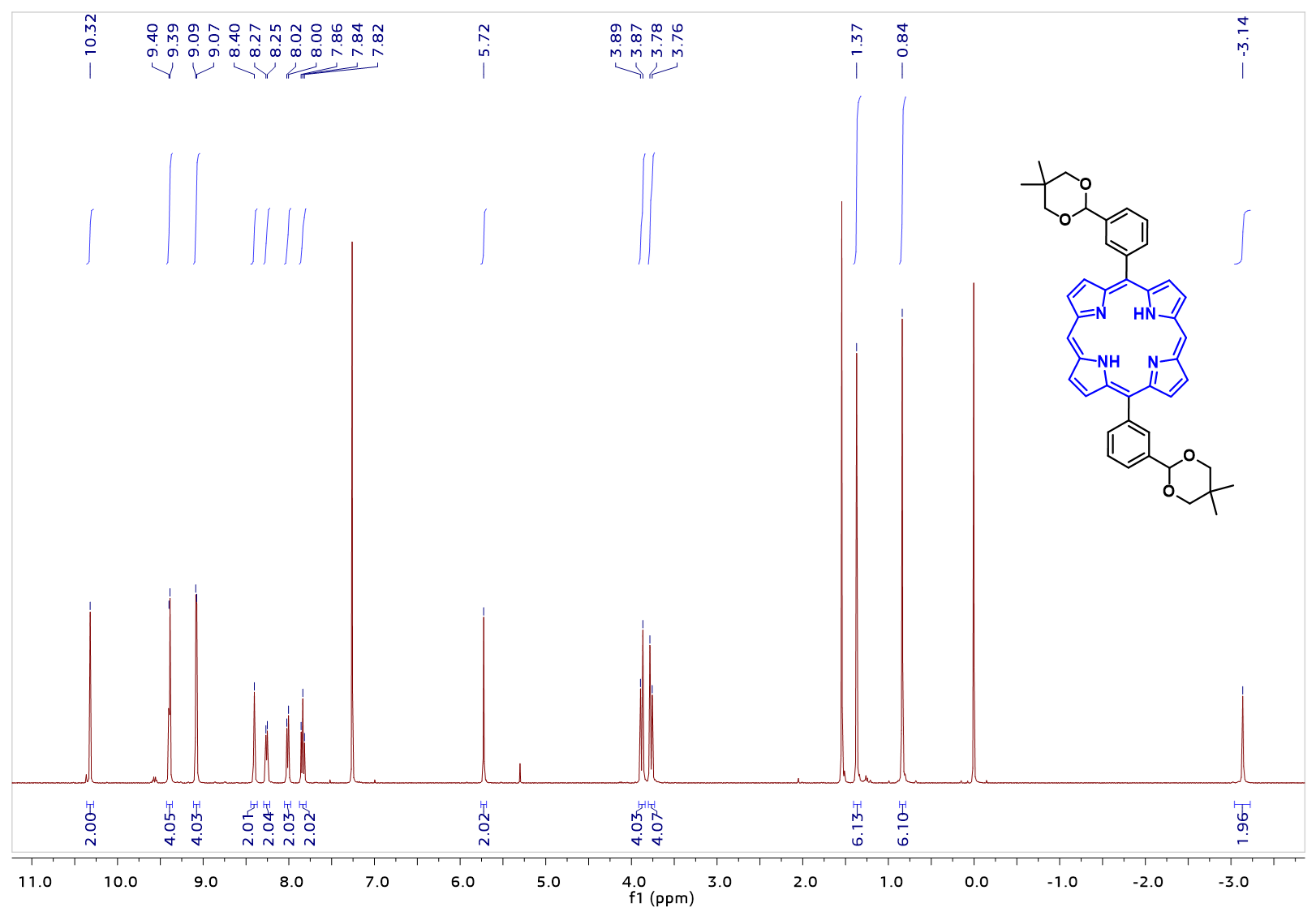


**Figure S3**. ^1^H NMR spectrum of 5,15-di((3-(1,3-dioxa-5,5-dimethylcyclohex-2-yl)phenyl)porphyrin


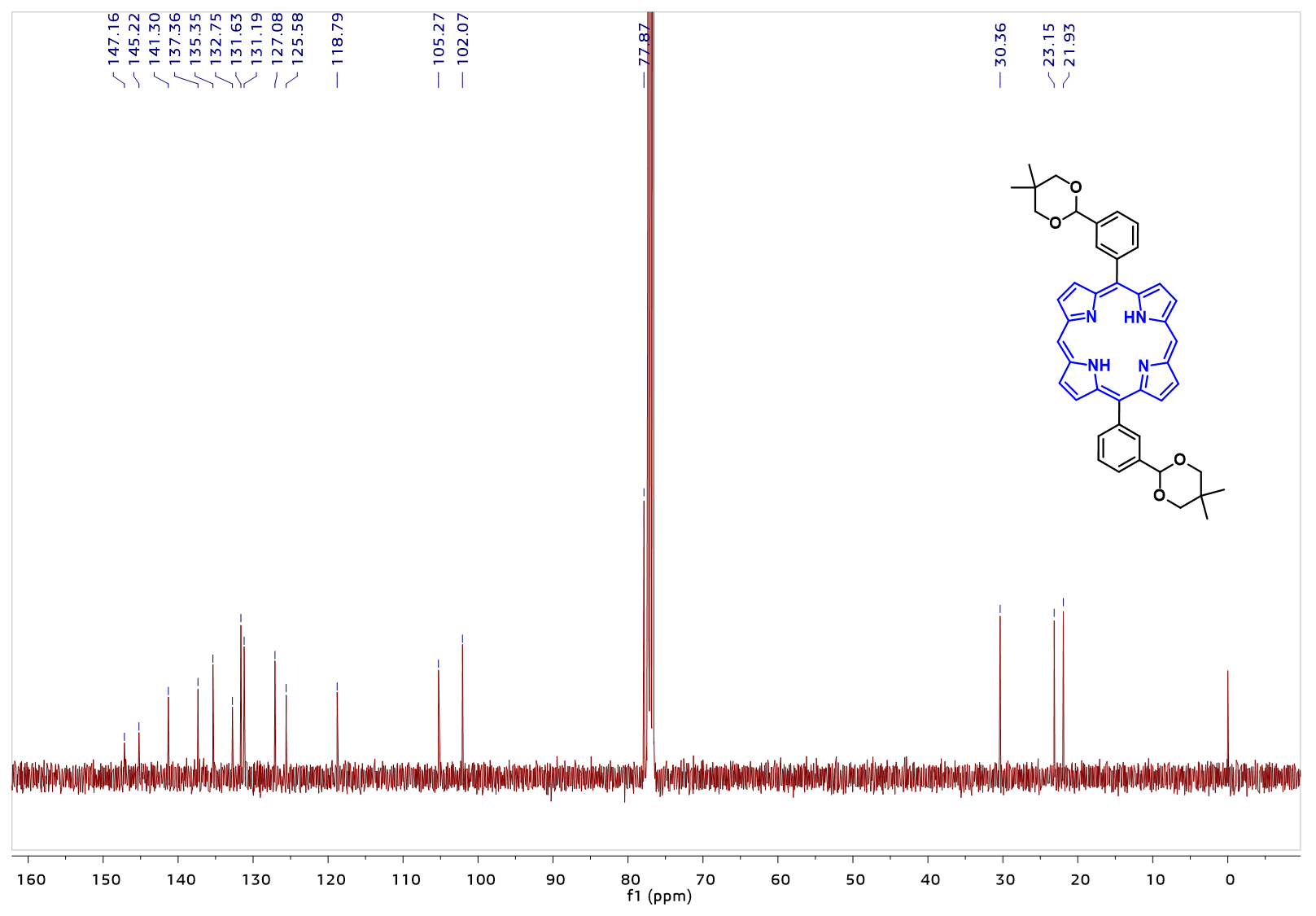


**Figure S4**. ^13^C NMR spectrum of 5,15-di((3-(1,3-dioxa-5,5-dimethylcyclohex-2-yl)phenyl)porphyrin

**Synthesis of** **5,15-di(3-benzaldehyde)porphyrin (*****m*-DBP-CHO)**

To a solution of 5,15-di((4-(1,3-dioxa-5,5-dimethylcyclohex-2-yl)phenyl)porphyrin (1.0 g, 1.45 mmol) in DCM (100 mL) and water (100 mL), trifluoroacetic acid (100 mL) was added and the green colored solution was stirred (protected from light) at room temperature for 24 h. The organic layer was separated, washed with water and dropped into 400 mL 0.4 M K_2_CO_3_ aqueous solution and methanol mixed solution (V: V=1:1). The residue was collected by filtration and then purified by column chromatography (DCM, silica gel, 200-300 mesh) to give a purple solid. Yield: 650.0 mg (86.6%). ^1^H NMR (400 MHz, CDCl_3_) *δ* 10.35 (s, 2H), 10.34 (s, 2H), 9.41 (d, *J* = 4.6 Hz, 2H), 9.00 (d, *J* = 4.6 Hz, 2H), 8.77 (s, 1H), 8.53 (d, *J* = 7.5 Hz, 1H), 8.37 (d, *J* = 7.8 Hz, 1H), 7.99 (t, *J* = 7.6 Hz, 1H), -3.15 (s, 2H). ^13^C NMR (400 MHz, CDCl_3_) *δ* 192.63, 146.89, 145.46, 142.42, 140.11, 135.47, 135.38, 135.30, 132.20, 130.73, 128.94, 128.88, 127.87, 117.35, 105.80. HR-MS (MALDI-TOF): m/z calculated for C_32_H_22_N_4_O_2_ 518.1743, found 519.1759 [M+H]^+^.


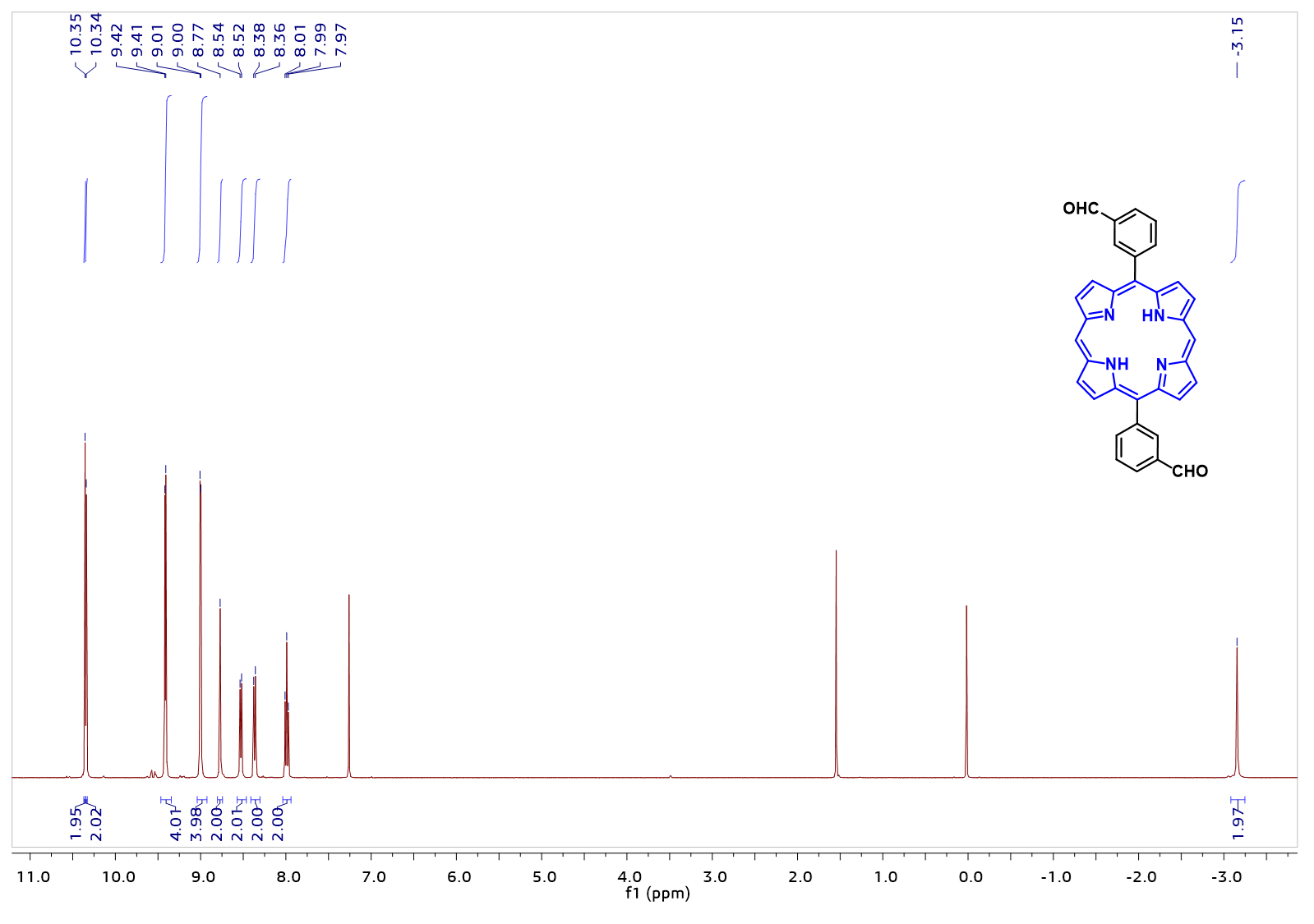


**Figure S5**. ^1^H NMR spectrum of *m*-DBP-CHO


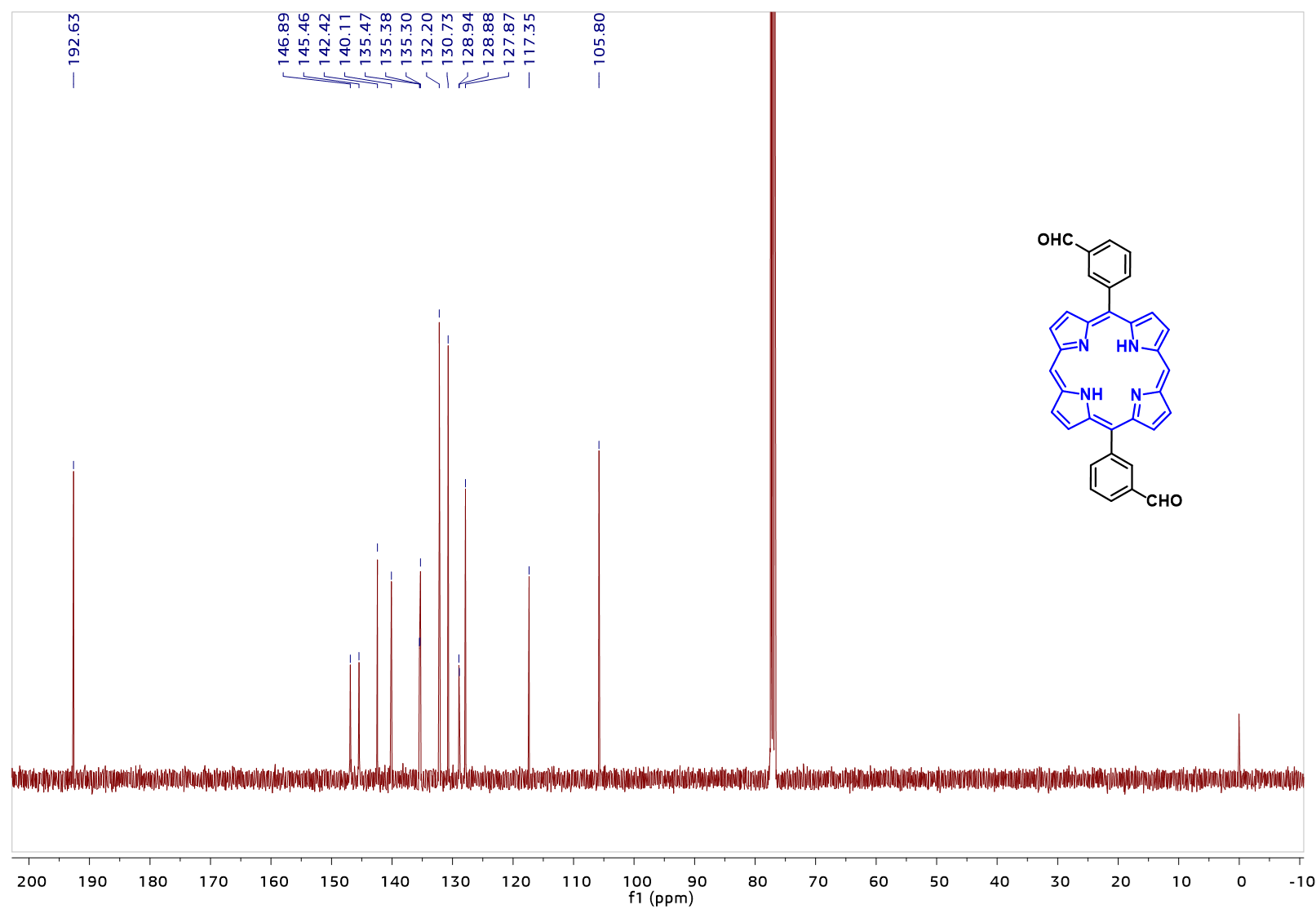


**Figure S6**. ^13^C NMR spectrum of *m*-DBP-CHO

**Synthesis of** **[5,15-di(3-benzaldehyde)porphyrinato]nickel(II) (*****m*-NiDBP-CHO)**

5,15-di(3-benzaldehyde)porphyrin (600.0 mg, 1.16 mmol) and nickel(**II**) acetate tetrahydrate (Ni(OAc)_2_•4H_2_O, 575.8 mg, 2.31 mmol) were added to a flask containing DMF (60 mL). The reaction mixture was heated under nitrogen atmosphere at 140 ºC for 4 h. After cooling to room temperature, the mixture was poured into 240 mL of water and methanol mixed solution (V: V=1: 1). The residue was collected by filtration and washed with water and methanol to yield product as a red solid. Yield: 650.0 mg (97.7%). ^1^H NMR (400 MHz, CDCl_3_) *δ* 10.29 (s, 2H), 9.95 (s, 2H), 9.19 (d, *J* = 4.6 Hz, 4H), 8.84 (d, *J* = 4.6 Hz, 4H), 8.57 (s, 2H), 8.32 (d, *J* = 6.5 Hz, 2H), 8.30 (d, *J* = 7.3 Hz, 2H), 7.91 (t, *J* = 7.5 Hz, 2H). ^13^C NMR (400 MHz, CDCl_3_) *δ* 192.53, 142.81, 142.66, 142.09, 139.15, 135.16, 134.58, 132.60, 132.06, 128.90, 127.76, 116.67, 105.58. HR-MS (MALDI-TOF): m/z calculated for C_32_H_20_N_4_O_2_Ni 574.0940, found 575.0975 [M+H]^+^.


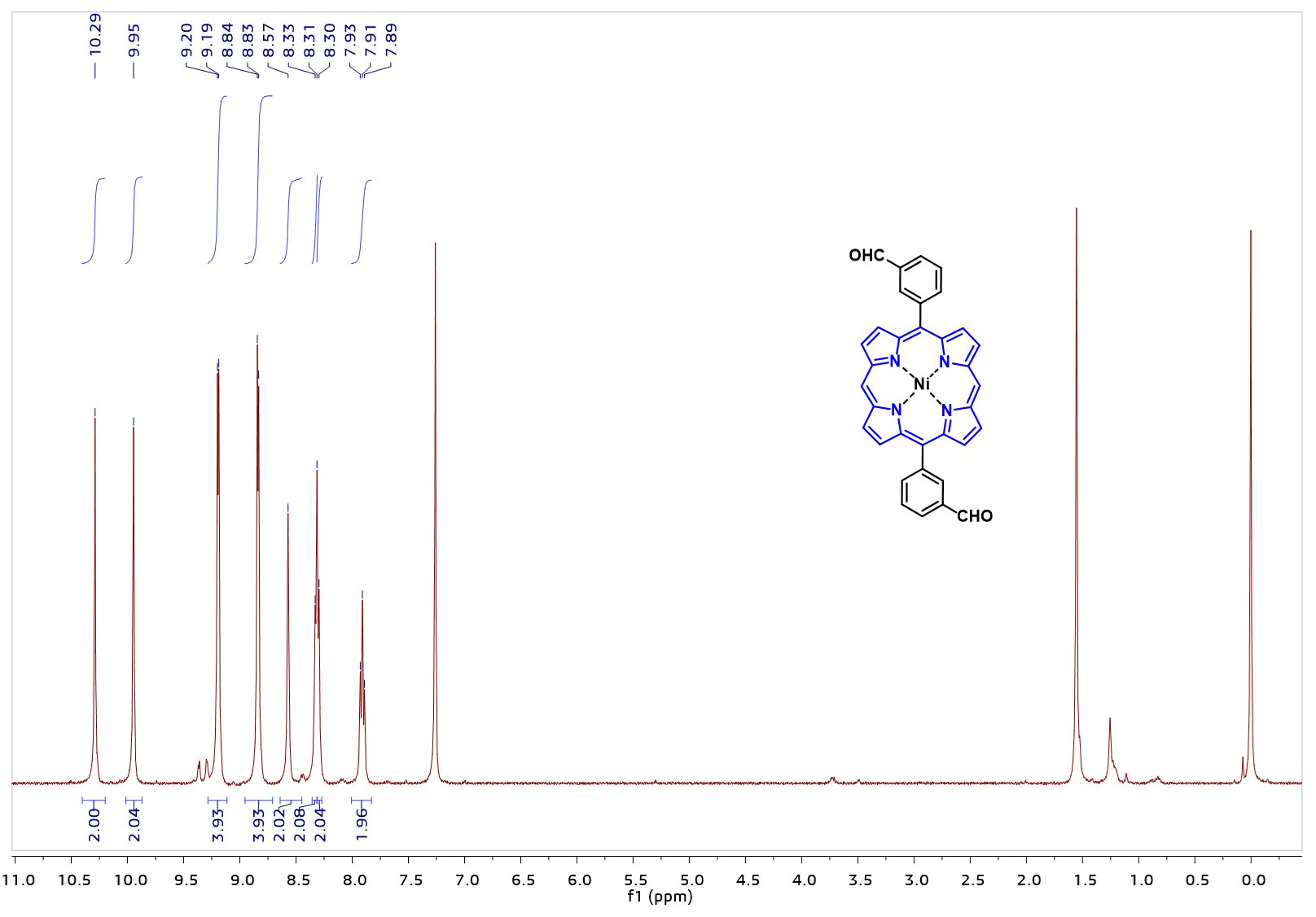


**Figure S7**. ^1^H NMR spectrum of *m*-NiDBP-CHO


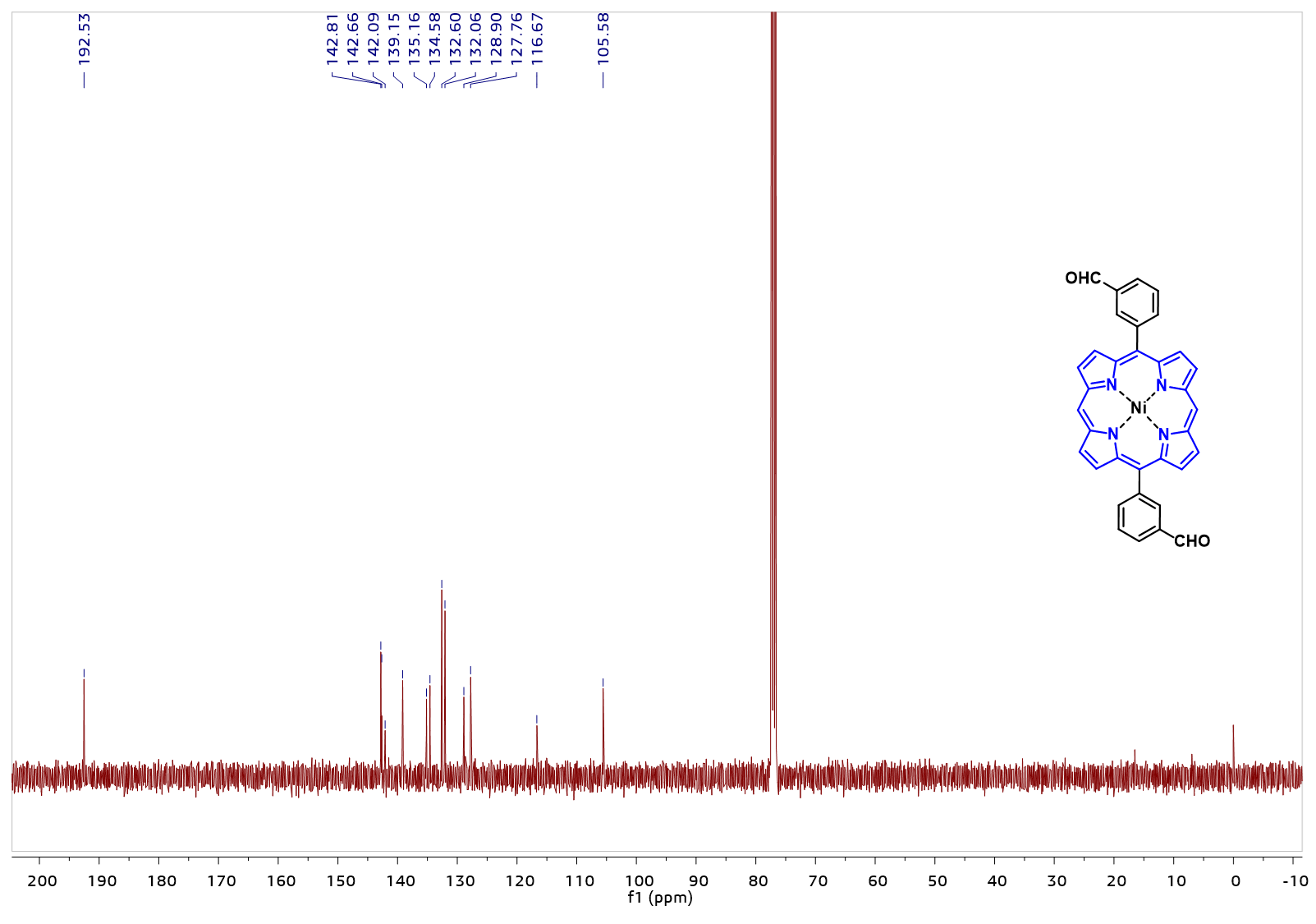


**Figure S8**. ^13^C NMR spectrum of *m*-NiDBP-CHO

**Synthesis of** ***m*-NiDBPA-COF**

***m*-NiDBP-CHO** (69.0 mg, 0.12 mmol) and TAPA (23.2 mg, 0.08 mmol) were added to 1,2-dichlorobenzene (*o*-DCB) and *n*-butanol (*n*-BuOH) (3 mL:3 mL) in a 10 mL Pyrex tube. The mixture was sonicated for 15 min and then 6 M aqueous acetic acid (0.6 mL) was added in the tube. The tube was flash-frozen at 77 K (liquid N_2_ bath) and evacuated. After three freeze-pump-thaw cycles, the system was then heated at 120 °C for 72 h, yielding a precipitate at the bottom of the tube, which was transferred to a centrifugal tube, washed with tetrahydrofuran (THF), and collected. Then, the solid was washed in a Soxhlet extractor with THF for 12 h and dried overnight at 80 °C in vacuo. The synthesis of ***m*-DBPA-COF** followed a similar procedure except that only ***m*-DBP-CHO** (66.2 mg, 0.12 mmol) was used as the monomer instead of ***m*-NiDBP-CHO**.

**3. Preparation of aziridines**

**Scheme S4**. Synthesis of aziridines

The typical procedure for the synthesis of aziridines is described as follows [2]: Firstly, bromine (0.075 mol, 12.0 g) in DCM (15 mL) was slowly dropped to a 15 mL DCM solution of dimethyl sulfide (0.075 mol, 4.7 g) in an ice bath over 30 min. The reaction mixture was then stirred for 3 h in an ice bath, and the orange crystals S1 were completely obtained and collected by filtration. Secondly, olefin (0.075 mol) was slowly dropped to a 75 mL CH_3_CN solution of S1 in an ice bath. The solution was stirred overnight after the addition of olefin. The white solid S2 was gradually generated during the process. Then, the crystals S2 were collected by filtration, and dried under vacuum. Thirdly, a solution of amine (0.15 mmol) was slowly added to a stirred solution of compound S2 in 55 mL water at room temperature. The reaction mixture was stirred for 24 h. The resulting mixture was then slowly dropped into 55 mL of saturated brine and extracted with DCM (3×20 mL). Finally, the solvent was dried with anhydrous Na_2_SO_4_ overnight and evaporated under reduced pressure to give aziridines as colorless liquids.

**1-Ethyl-2-phenylaziridine**

^1^H NMR (400 MHz, CDCl_3_) *δ* 7.31 - 7.20 (m, 5H), 2.55 - 2.42 (m, 2H), 2.31 (dd, *J* = 6.5, 3.3 Hz, 1H), 1.90 (d, *J* = 3.3 Hz, 1H), 1.65 (d, *J* = 6.6 Hz, 1H), 1.20 (t, *J* = 7.1 Hz, 3H).


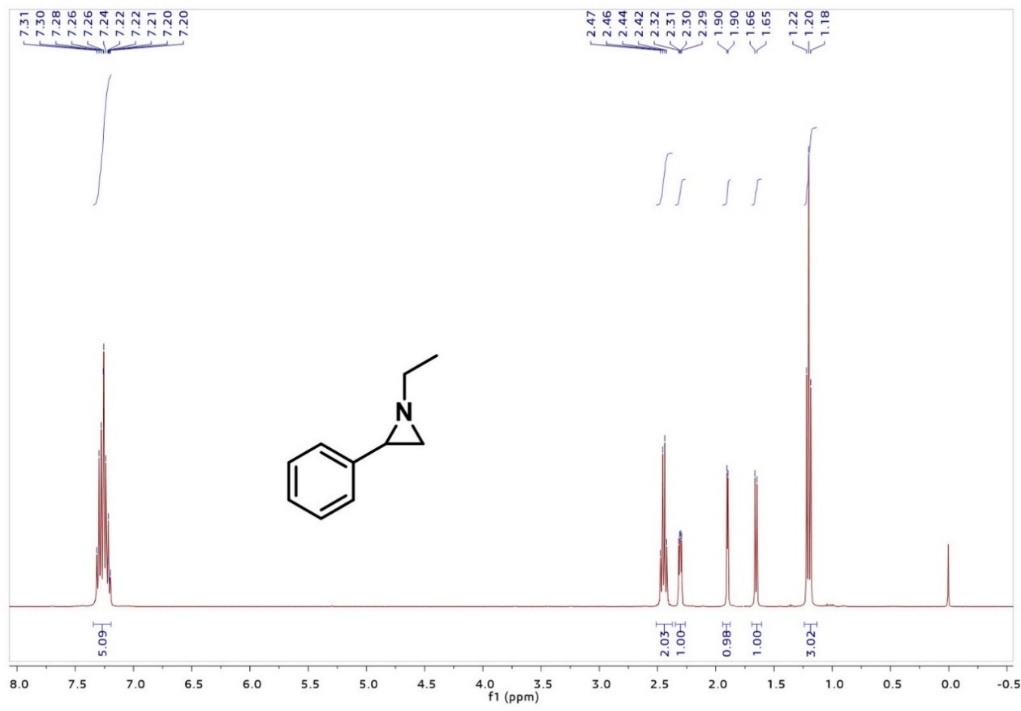


**Figure S9**. ^1^H NMR spectrum of 1-ethyl-2-phenylaziridine

**1-Propyl-2-phenylaziridine**

^1^H NMR (400 MHz, CDCl_3_) *δ* 7.31 - 7.19 (m, 5H), 2.48 (dt, *J* = 11.5, 7.3 Hz, 1H), 2.35 - 2.22 (m, 2H), 1.89 (d, *J* = 3.3 Hz, 1H), 1.70 - 1.65 (m, 2H), 1.64 - 1.59 (m, 1H), 0.96 (t, *J* = 7.4 Hz, 3H).


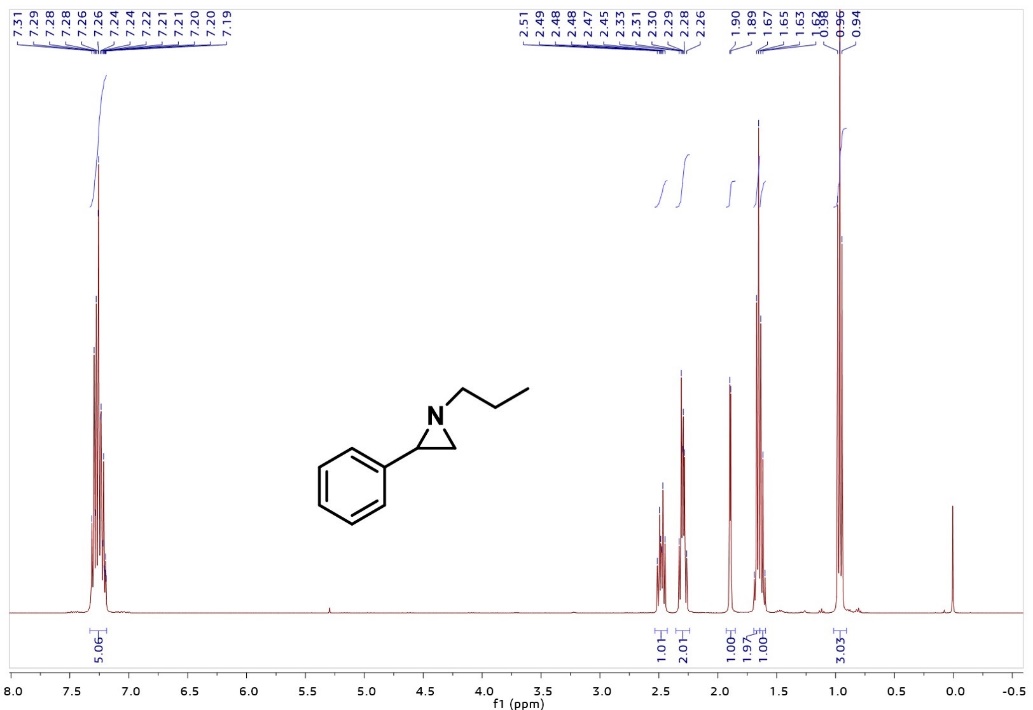


**Figure S10**. ^1^H NMR spectrum of 1-propyl-2-phenylaziridine

**1-Butyl-2-phenylaziridine**

^1^H NMR (400 MHz, CDCl_3_) δ 7.32 - 7.20 (m, 5H), 2.50 (dt, *J* = 11.5, 7.3 Hz, 1H), 2.40 - 2.23 (m, 2H), 1.89 (d, *J* = 3.3 Hz, 1H), 1.66 (d, *J* = 6.6 Hz, 1H), 1.63 - 1.57 (m, 2H), 1.47 - 1.33 (m, 2H), 0.92 (t, *J* = 7.3 Hz, 3H).


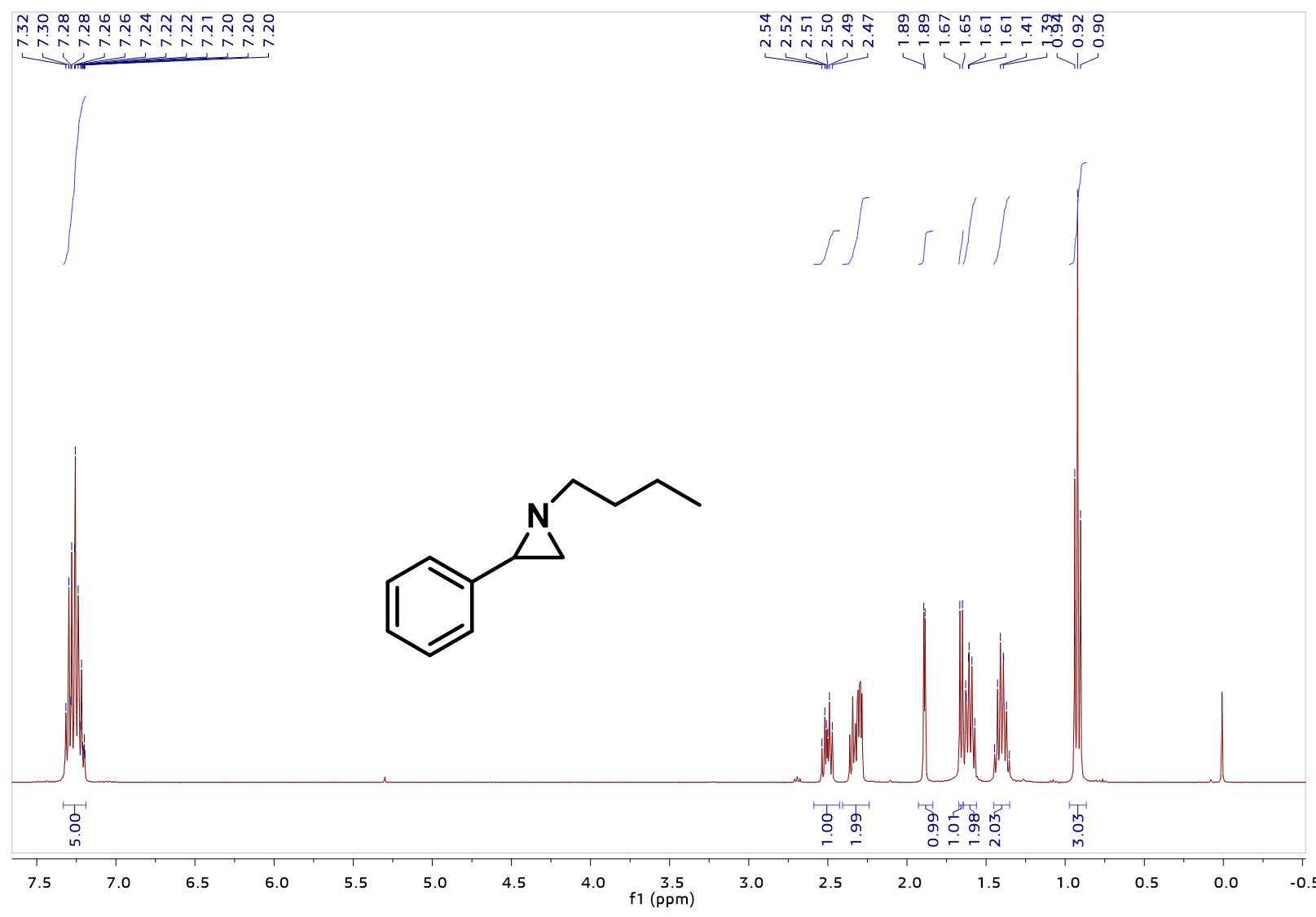


**Figure S11**. ^1^H NMR spectrum of 1-butyl-2-phenylaziridine

**1-Amyl-2-phenylaziridine**

^1^H NMR (400 MHz, CDCl_3_) *δ* 7.31- 7.20 (m, 5H), 2.47 (dt, *J* = 11.5, 7.4 Hz, 1H), 2.38 - 2.24 (m, 2H), 1.89 (d, *J* = 3.2 Hz, 1H), 1.65 (d, *J* = 6.6 Hz, 1H), 1.64 - 1.57 (m, 2H), 1.38 - 1.27 (m, 4H), 0.89 (t, *J* = 8.7, 5.3 Hz, 3H).


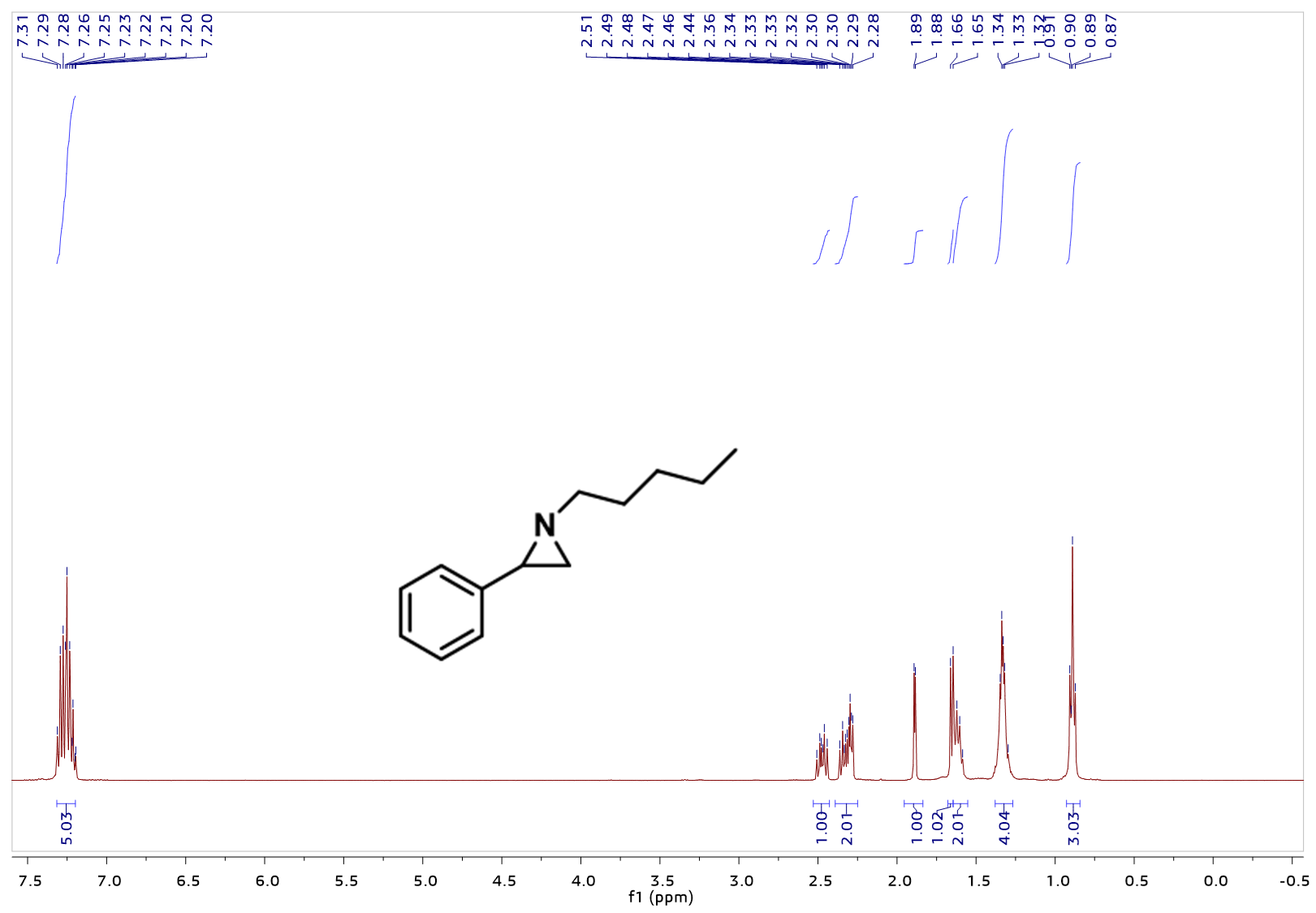


**Figure S12**. ^1^H NMR spectrum of 1-amyl-2-phenylaziridine

**2-(4-Methphenyl)-1-ethylaziridine**

^1^H NMR (400 MHz, CDCl_3_) *δ* 7.12 (q, *J* = 8.2 Hz, 4H), 2.51 - 2.36 (m, 2H), 2.32 (s, 3H), 2.27 (dd, *J* = 6.5, 3.3 Hz, 1H), 1.87 (d, *J* = 3.3 Hz, 1H), 1.63 (d, *J* = 6.5 Hz, 1H), 1.19 (t, *J* = 7.1 Hz, 3H).


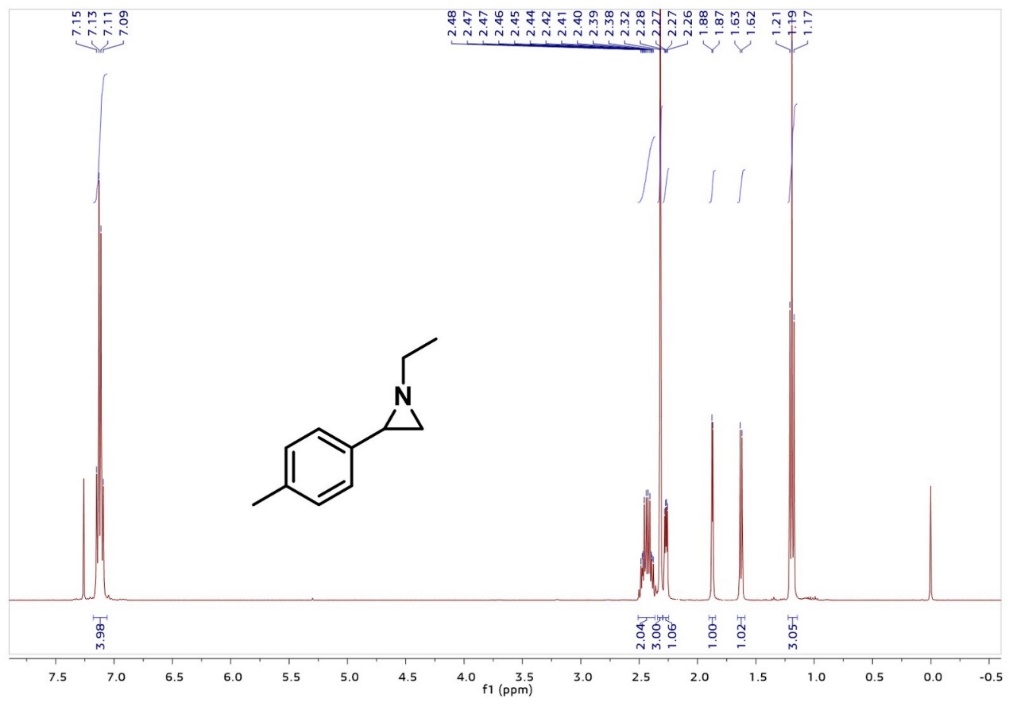


**Figure S13**. ^1^H NMR spectrum of 2-(4-methphenyl)-1-ethylaziridine

**2-(4-Methoxyphenyl)-1-ethylaziridine**

^1^H NMR (400 MHz, CDCl_3_) *δ* 7.19 (d, *J* = 8.6 Hz, 2H), 6.86 (d, *J* = 8.7 Hz, 2H), 2.51 – 2.42 (m, 3H), 2.28 (dd, *J* = 6.5, 3.4 Hz, 2H), 1.88 (d, *J* = 3.3 Hz, 1H),1.88 (d, *J* = 3.3 Hz, 1H) 1.63 (d, *J* = 6.5 Hz, 1H), 1.21 (t, *J* = 7.1 Hz, 3H).


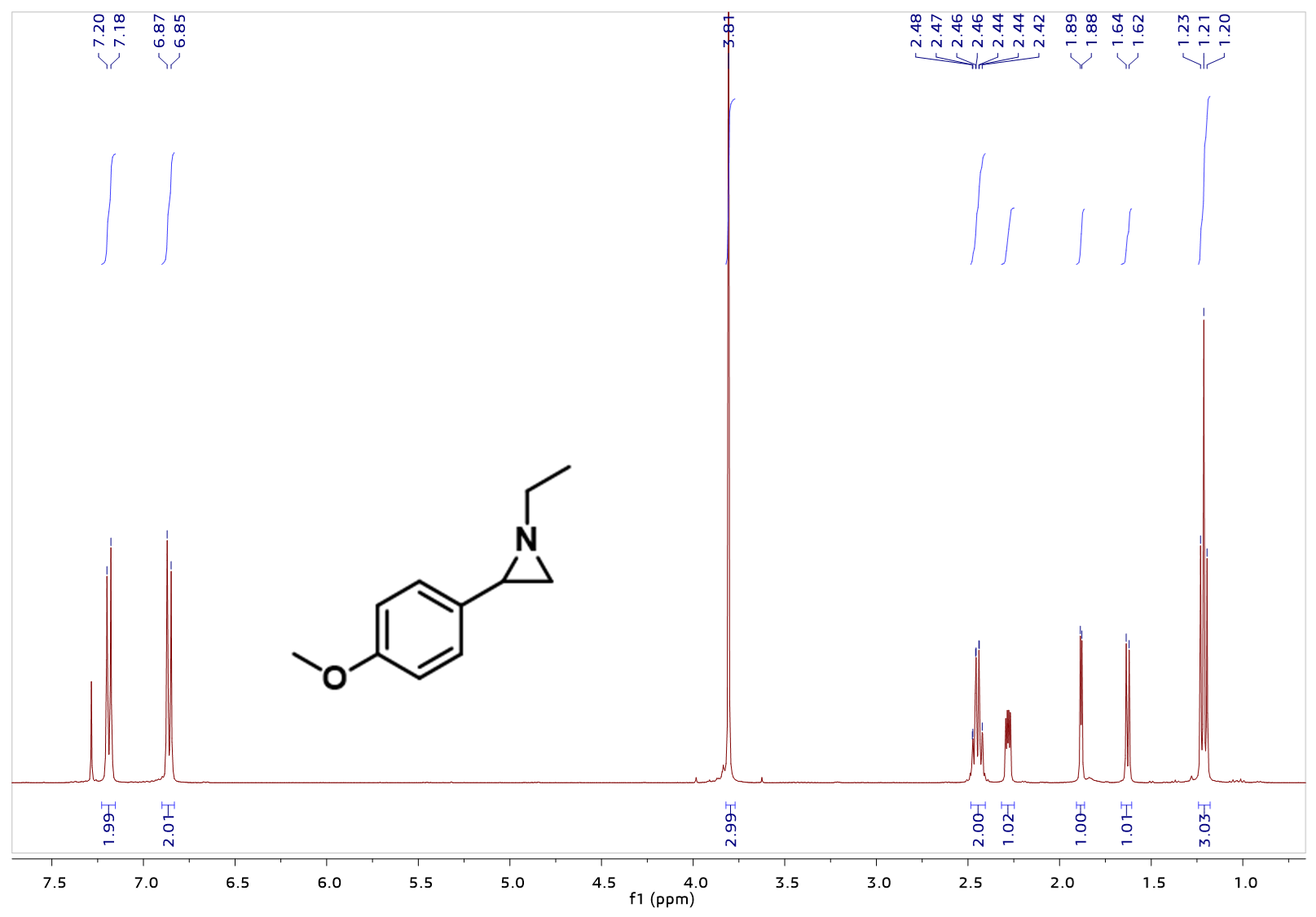


**Figure S14**. ^1^H NMR spectrum of 2-(4-methoxyphenyl)-1-ethylaziridine

**2-(4-****Fuorophenyl)-1-ethylaziridine**

^1^H NMR (400 MHz, CDCl_3_) *δ* 7.20 (dd, *J* = 8.5, 5.5 Hz, 2H), 6.97 (t, *J* = 8.7 Hz, 2H), 2.43 (q, *J* = 6.9 Hz, 2H), 2.28 (dd, *J* = 6.4, 3.3 Hz, 1H), 1.85 (d, *J* = 3.3 Hz, 1H), 1.64 (d, *J* = 6.5 Hz, 1H), 1.19 (t, *J* = 7.1 Hz, 3H).


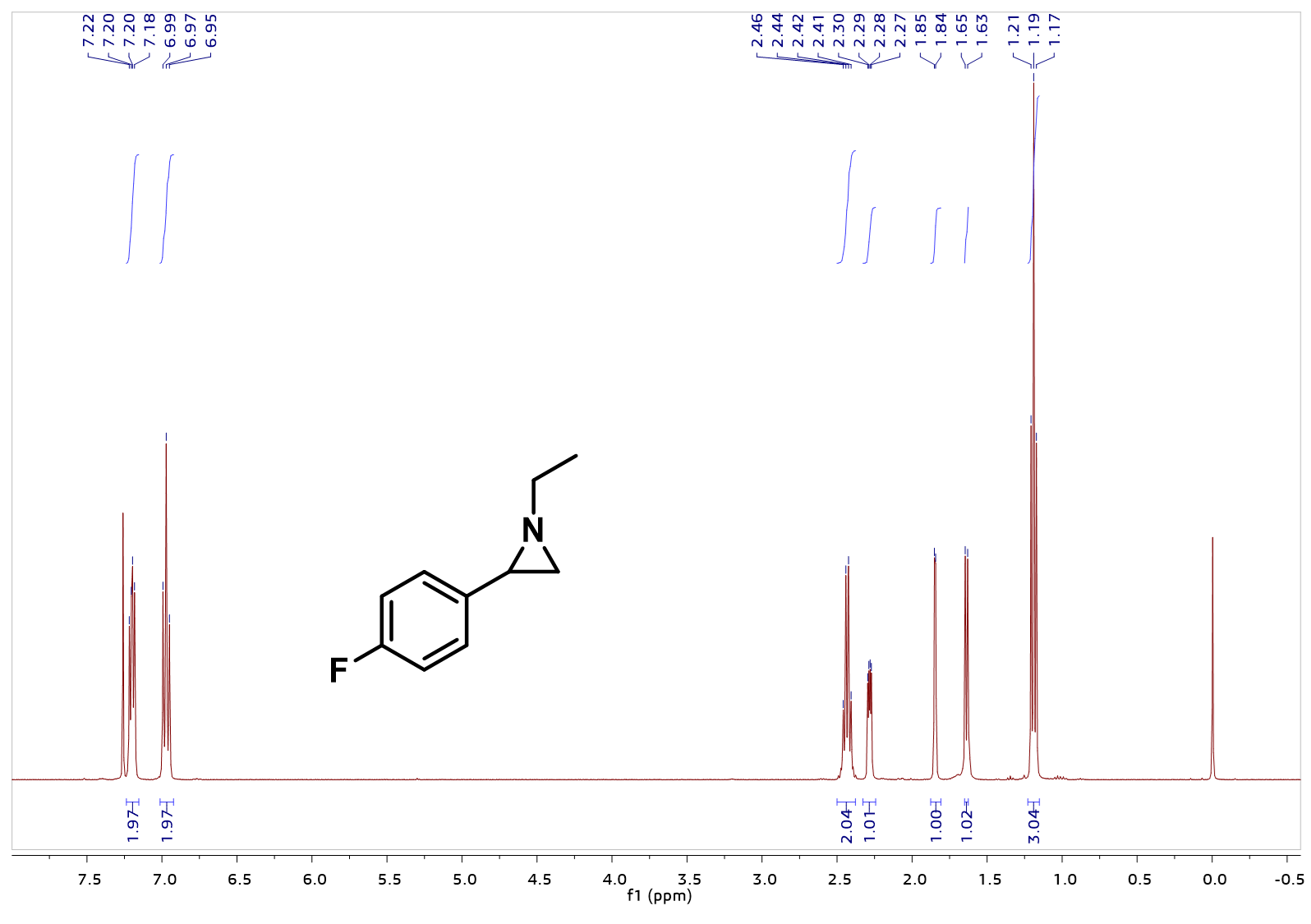


**Figure S15**. ^1^H NMR spectrum of 2-(4-fuorophenyl)-1-ethylaziridine

**2-(4-Chlorophenyl)-1-ethylaziridine**

^1^H NMR (400 MHz, CDCl_3_) *δ* 7.25 (d, *J* = 8.3 Hz, 2H), 7.17 (d, *J* = 8.5 Hz, 2H), 2.43 (q, *J* = 7.1 Hz, 2H), 2.27 (dd, *J* = 6.5, 3.3 Hz, 1H), 1.84 (d, *J* = 3.3 Hz, 1H), 1.65 (d, *J* = 6.5 Hz, 1H), 1.18 (t, *J* = 7.1 Hz, 3H).


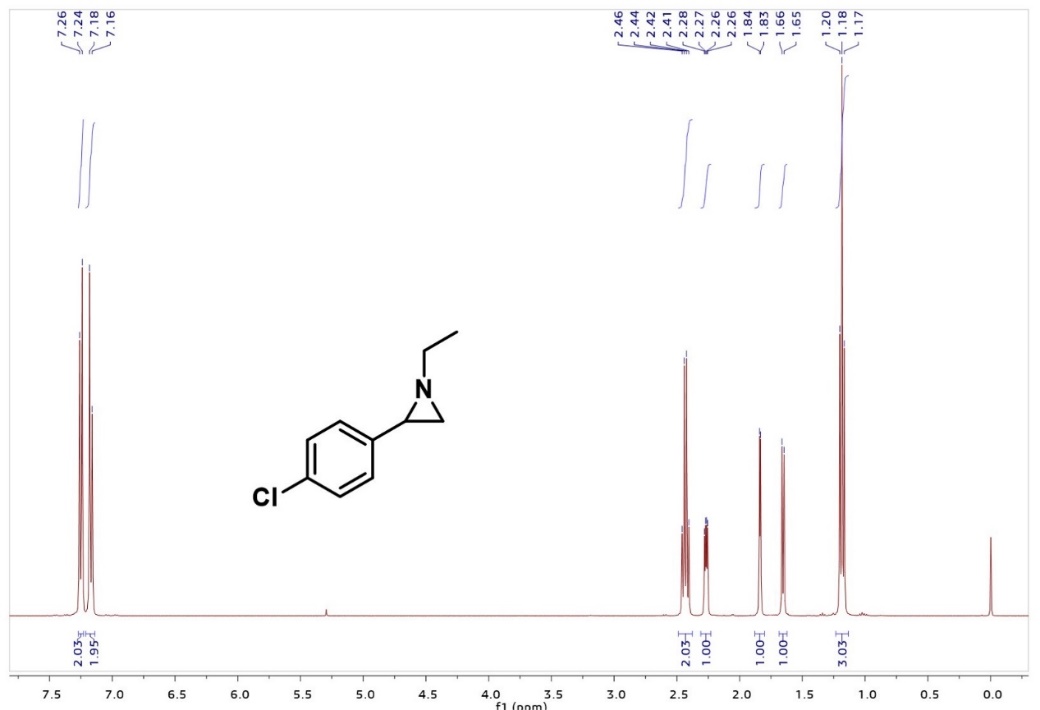


**Figure S16**. ^1^H NMR spectrum of 2-(4-chlorophenyl)-1-ethylaziridine

**2-(4-Bromophenyl)-1-ethylaziridine**
^1^H NMR (400 MHz, CDCl_3_) *δ* 7.40 (d, *J* = 8.4 Hz, 2H), 7.12 (d, *J* = 8.4 Hz, 2H), 2.43 (q, *J* = 7.1 Hz, 2H), 2.26 (dd, *J* = 6.5, 3.2 Hz, 1H), 1.84 (d, *J* = 3.2 Hz, 1H), 1.66 (d, *J* = 6.5 Hz, 1H), 1.18 (t, *J* = 7.1 Hz, 3H).


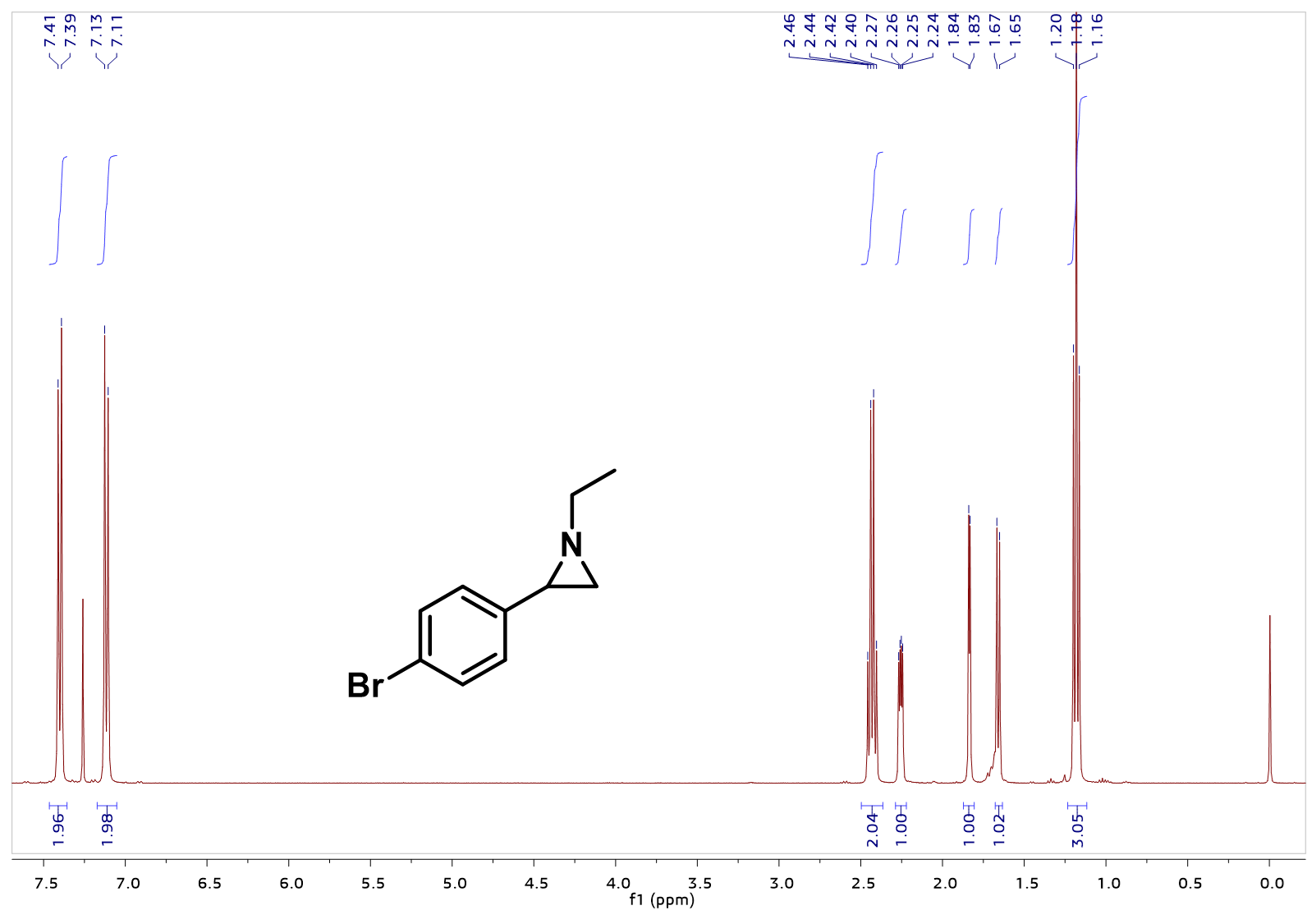


**Figure S17**. ^1^H NMR spectrum of 2-(4-bromophenyl)-1-ethylaziridine

**4. Catalytic CO_2_ cycloaddition reaction**

A general catalytic reaction was conducted in 10 mL Schlenk tube using aziridine (2 mmol) in a solvent free environment at a 1 atm CO_2_ atmosphere with catalyst (0.5 mol% porphyrin centers/metal ions) and TBAB (32.2 mg, 5 mol%) co-catalyst stirring at 500 min^−1^ for 48 h. Before the reaction, the tube was flash-frozen at 77 K (liquid N_2_ bath), evacuated and then sealed with a CO_2_ balloon. The photocatalytic reaction was conducted under irradiation with a 300 W Xe lamp, and the reaction temperature maintained at approximately 30°C. After catalyst separation by centrifugation, the conversion of the reactant was determined by ^1^H NMR spectroscopy in CDCl_3_. For the recycling test, the catalysts were separated by centrifuge, washed by tetrahydrofuran, dried at 80 ℃ for 12 h, and then reused for the next run.

**5. Characterizations**

**5.1 Structure characterization**

**Table S1**. Crystalline cell parameters for the AA-stacking mode of *m*-DBPA-COF and *m*-NiDBPA-COF.

| *m*-DBPA-COF | *m*-NiDBPA-COF |
| --- | --- |
| a=48.7997 Å, b=42.7714 Å, c=3.7890 Å | a=48.7455 Å, b=42.7189 Å, c=3.7844 |
| α=100.8482 °, β=79.9655 °, γ=124.2181 ° | α=100.8476 °, β=79.9687 °, γ=124.2182 ° |


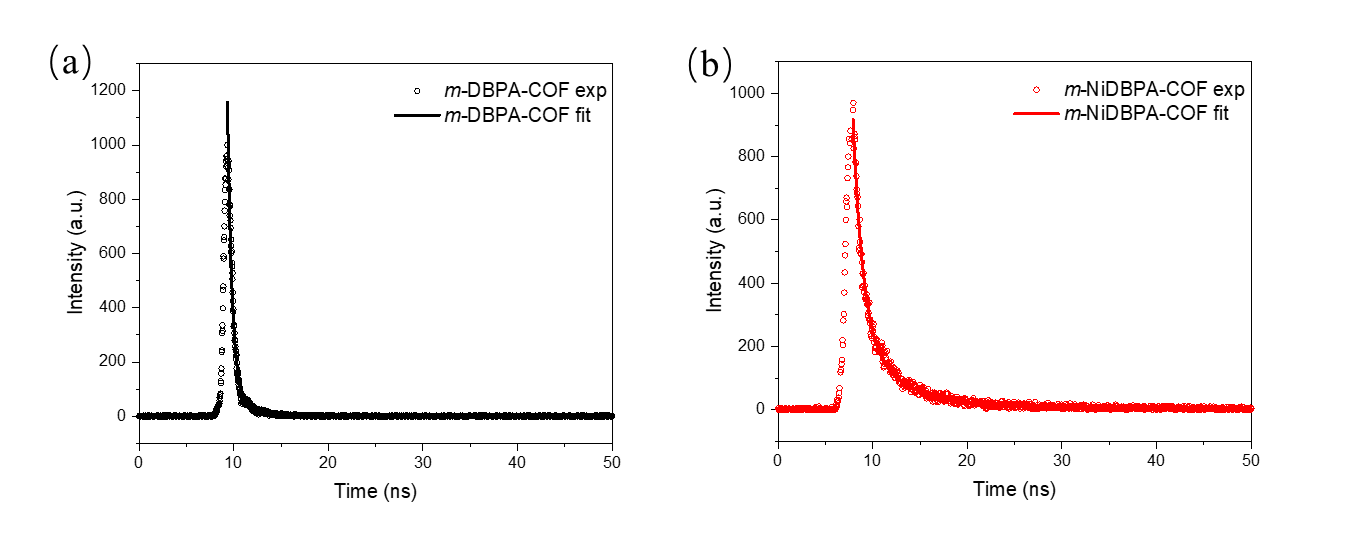


**Figure S18**. Time-resolved photoluminescence spectra of (a) *m*-DBPA-COF, (b) *m*-NIDBPA-COF at an excitation wavelength of 375 nm.

**Table** **S2**. Fitted parameters of time-resolved PL spectra.

| **Sample** | **Component** | **Lifetime (ns)** | **Intensity (%)** | **Decay Lifetime (τ_ave_) (ns)** |
| --- | --- | --- | --- | --- |
| *m*-DBPA-COF | τ_1_ | 0.4859 | 77.09 | 0.7684 |
|  | τ_2_ | 1.7188 | 22.91 |  |
| *m*-NiDBPA-COF | τ_1_ | 1.1498 | 48.61 | 3.2223 |
|  | τ_2_ | 5.1826 | 51.39 |  |

**Table S3**. The kinetic parameters of photocatalyst electrodes.

| **Sample** | **Rs (Ω)** | **Rct (Ω)** |
| --- | --- | --- |
| *m*-DBPA-COF | 59.95 | 34819 |
| *m*-NiDBPA-COF | 64.45 | 26059 |

**5.2 Catalytic performance of *m*-NiDBPA-COF for CO_2_ cycloaddition to aziridines**


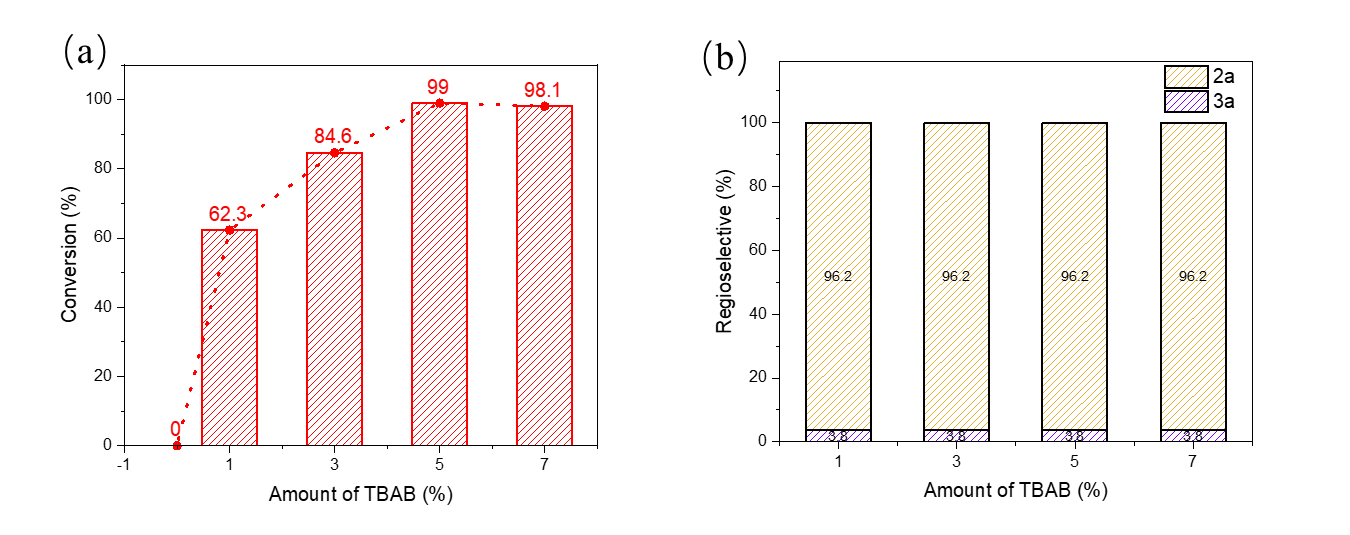


**Figure S19**. Effect of TBAB amount. Reaction conditions: 1-ethyl-2-phenylaziridine (2.0 mmol), *m*-NiDBPA-COF (7.2 mg, 0.5 mol% Ni ions), 1 atm CO_2_, 300 W Xenon lamp, reaction time to 48 h. The conversion and regioselectivity were determined by ^1^H NMR.


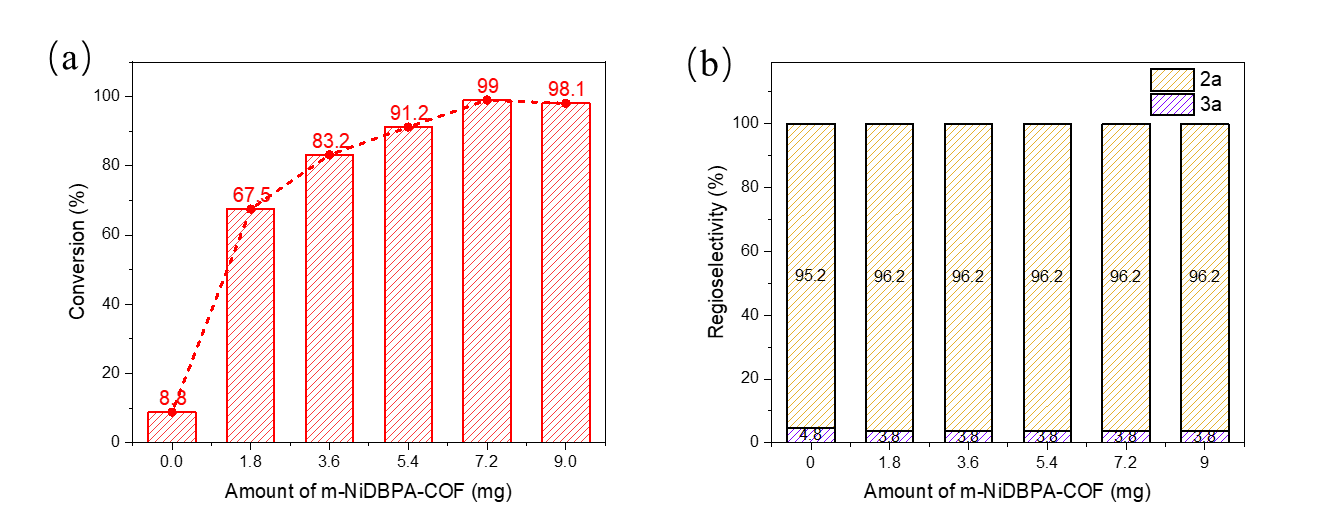


**Figure S20**. Effect of *m*-NiDBPA-COF loading. Reaction conditions: 1-ethyl-2-phenylaziridine (2.0 mmol), TBAB (32.2 mg, 5 mol%), 1 atm CO_2_, 300 W Xenon lamp, reaction time to 48 h. The conversion and regioselectivity were determined by ^1^H NMR.

**Figure S21**. Effect of reaction time. Reaction conditions: 1-ethyl-2-phenylaziridine (2 mmol), *m*-NiDBPA-COF (7.2 mg, 0.5 mol% Ni ions), TBAB (32.2 mg, 5 mol%), 1 atm CO_2_. 300 W Xenon lamp. The conversion was determined by ^1^H NMR.


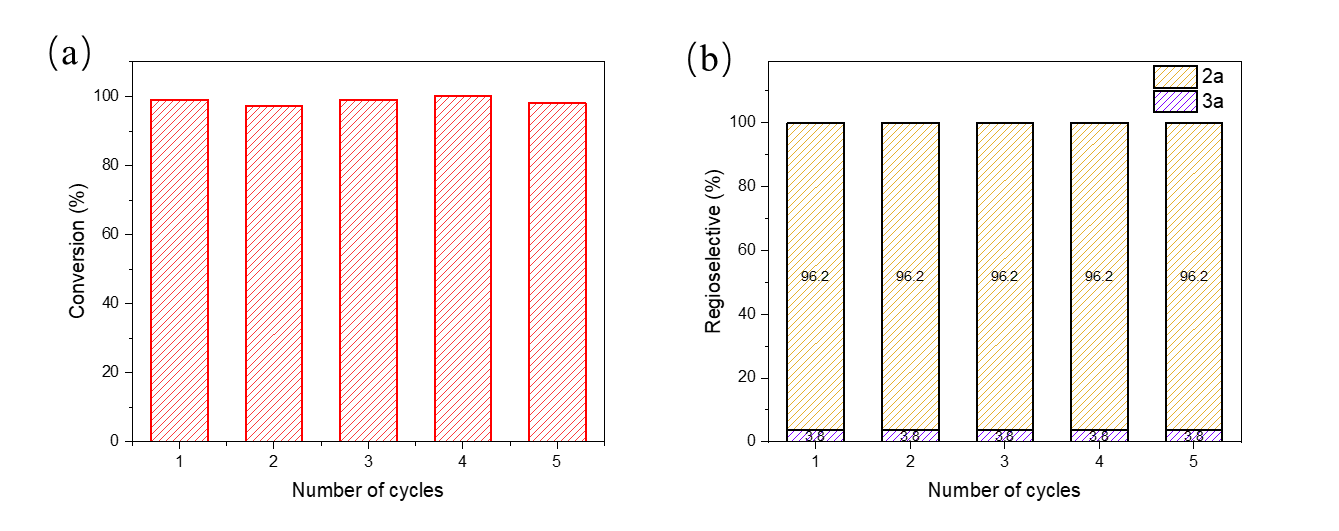


**Figure S22**. The recyclability of *m*-NiDBPA-COF.


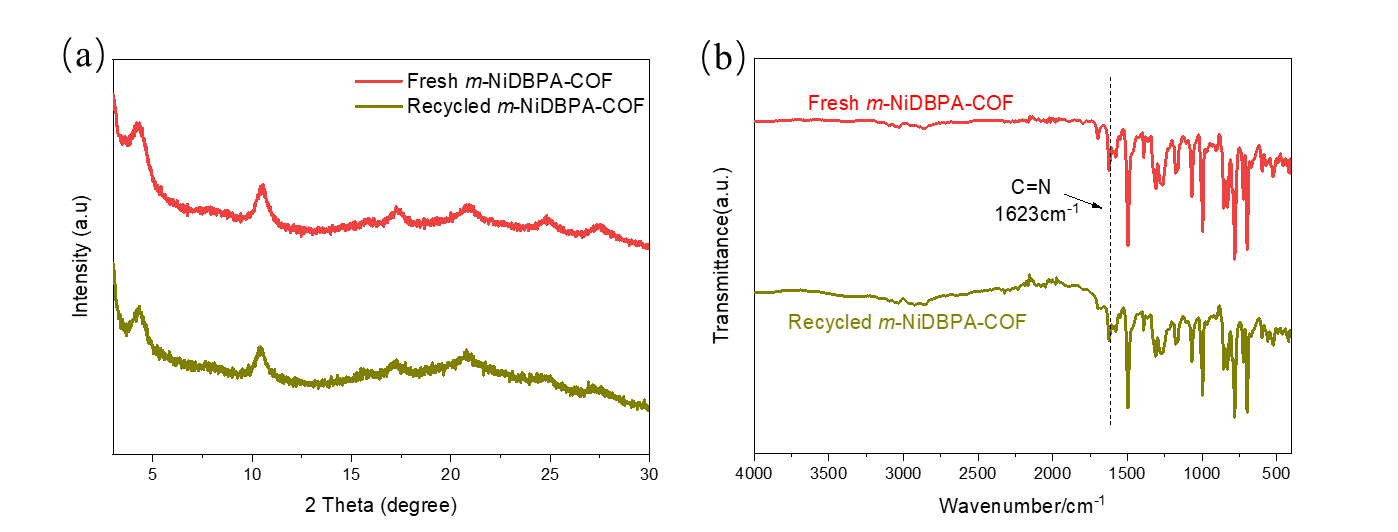


**Figure S23**. (a) PXRD spectra of *m*-NiDBPA-COF before and after 5 cycles of the reaction; (b) FTIR spectra of *m*-NiDBPA-COF before and after 5 cycles of the reaction.

**Table S4**. Chemical shifts (*δ*, ppm) for the integrated protons in the aziridines and corresponding oxazolidinones (in CDCl_3_).

|  | aziridine | *δ*H (^1^Hc) | *δ*H (^1^Hc1) | *δ*H (^1^Hc2) |
| --- | --- | --- | --- | --- |
| 1 |  | 1.90 | 3.93 | 4.61 |
| 2 |  | 1.89 | 3.91 | 4.61 |
| 3 |  | 1.88 | 3.91 | 4.61 |
| 4 |  | 1.89 | 3.91 | 4.61 |
| 5 |  | 1.86 | 3.89 | 4.59 |
| 6 |  | 1.85 | 3.87 | 4.57 |
| 7 |  | 1.84 | 3.91 | 4.60 |
| 8 |  | 1.84 | 3.92 | 4.60 |
| 9 |  | 1.84 | 3.92 | 4.60 |

**Determination of the aziridine substrates conversion**


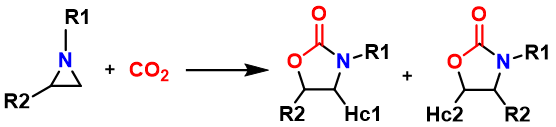


For each reaction, the conversion of aziridine and the regioselectivity of oxazolidinone were determined by comparison of the ^1^H NMR integrals of the corresponding protons in the starting material (^1^Hc) and in the product (^1^Hc_1_ and ^1^Hc_2_) according to equation (1), equation (2) and Table S4. The reaction rate of photocatalytic CO_2_ cycloaddition was furether calculated accrding to equation (3). Detailed spectra of reaction mixtures have been shown in Figure S24-S32 below.

$Conversion=\frac{{}^{1}{Hc1+}{}^{1}{Hc2}}{({}^{1}{Hc1+{}^{1}{Hc2)+}}{}^{1}{Hc}}\times100\%$ (1)

*Regioselectivity =*$\frac{{}^{1}{Hc1}}{{}^{1}{Hc1+}{}^{1}{Hc2}}\times100\%$ : $\frac{{}^{1}{Hc2}}{{}^{1}{Hc1+}{}^{1}{Hc2}}\times100\%$ (2)

$Reaction Rate=\frac{Conversion*n(aziridines)}{n\left( photocatalyst \right)*Reaction Time}$ (3)

**Table S5.** Catalytic performance of *m*-NiDBPA-COF over various aziridines.^a^

|  | | | |
| --- | --- | --- | --- |
|  | aziridine | Conversion/%^b^ | Regio-sel^b,c^ |
| 1 |  | 99.0 | 96.2: 3.8 |
| 2 |  | 95.4 | 96.2: 3.8 |
| 3 |  | 91.4 | 94.3: 5.7 |
| 4 |  | 88.2 | 95.2: 4.8 |
| 5 |  | 95.4 | 97.1: 2.9 |
| 6 |  | >99 | 99.0: 1.0 |
| 7 |  | 73.9 | 95.2: 4.8 |
| 8 |  | 77.6 | 96.2: 3.8 |
| 9 |  | 83.2 | 96.2:3.8 |

^a^ Reaction conditions: 1-ethyl-2-phenylaziridine (2 mmol), *m*-NiDBPA-COF (7.2 mg, 0.5 mol% Ni ions), TBAB (32.2 mg, 5 mol%), 1 atm CO_2_, 300 W Xenon lamp, reaction time to 48 h. ^b^ The total yield of the conversion and regioselectivity was determined by ^1^H NMR. ^c^ Molar ratio of 2 to 3.

**Table S6.** Comparison of the catalytic performance of different nucleophilic reagents.^a^

|  | | | |  |
| --- | --- | --- | --- | --- |
|  | Cocatalyst | Conversion/%^b^ | Regio-sel^b,c^ | |
| 1 | TBAC (27.8mg, 5 mol%) | 49.3 | 98.0: 2.0 | |
| 2 | TBAB (32.2 mg, 5 mol%) | 99.0 | 96.2: 3.8 | |
| 3 | TBAI (36.9 mg, 5 mol%) | 3.6 | 95.2: 4.8 | |

^a^ Reaction conditions: 1-ethyl-2-phenylaziridine (2 mmol), *m*-NiDBPA-COF (7.2 mg, 0.5 mol% Ni ions), 1 atm CO_2_, 300 W Xenon lamp, reaction time to 48 h. ^b^ The total yield of the conversion and regioselectivity was determined by ^1^H NMR. ^c^ Molar ratio of 2 to 3.

To evaluate the influence of the different nucleophiles on catalytic performance, tetrabutylammonium chloride (TBAC) and tetrabutylammonium iodide (TBAI) were also employed as cocatalysts to accelerate the reaction. The catalytic activities were observed in the following order: TBAB > TBAC > TBAI. Using TBAI (36.9 mg, 5 mol%) in place of TBAB as the cocatalyst resulted in a dramatic reduction in the conversion of 1-ethyl-2-phenylaziridine to negligible levels. This reduction was likely attributable to the photosensitivity of TBAI, which rendered it unstable when exposed to light.


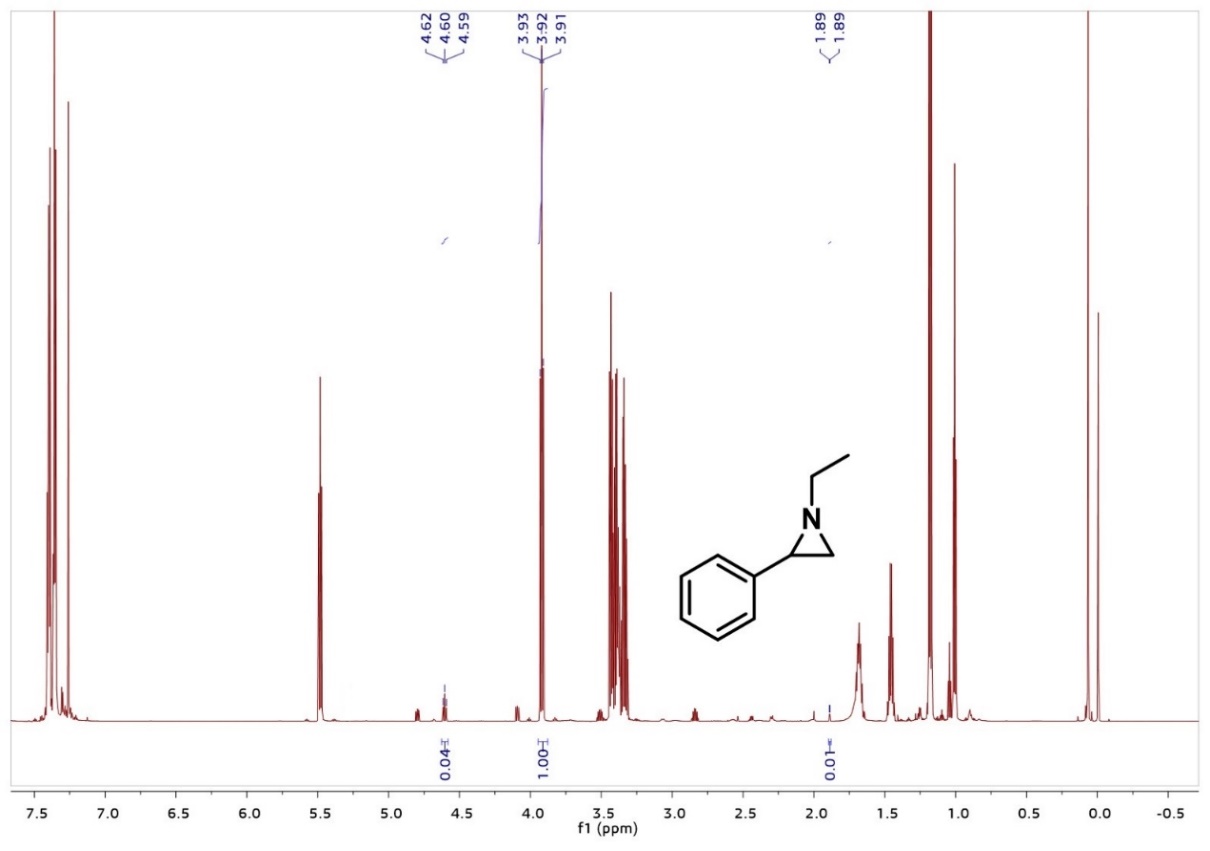


**Figure S24**. ^1^H NMR (400 MHz, CDCl_3_) spectrum of reaction system in Figure 4g entry 1. Conversion=(1+0.04)/(1+0.04+0.01)×100%=99.0%.


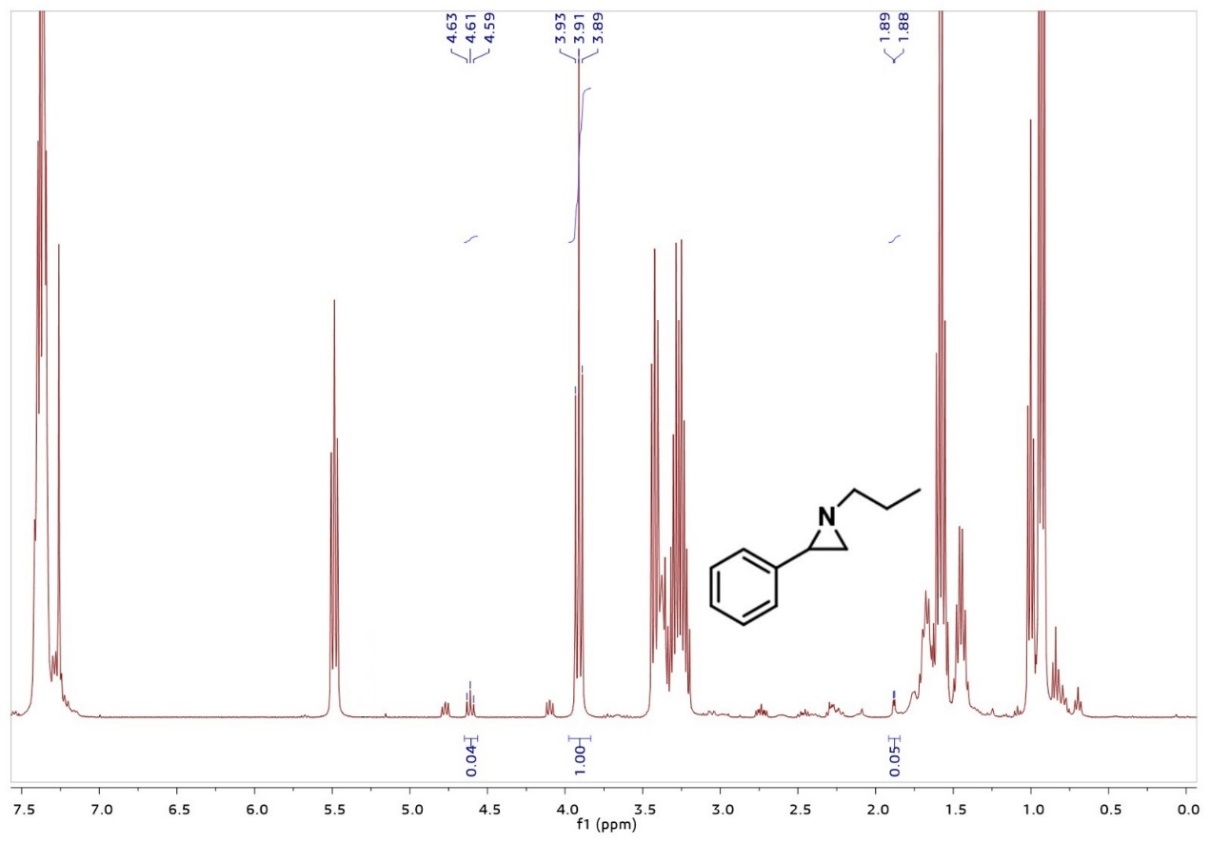


**Figure S25**. ^1^H NMR (400 MHz, CDCl_3_) spectrum of reaction system in Figure 4g entry 2. Conversion=(1+0.04)/(1+0.04+0.05)×100%=95.4%.


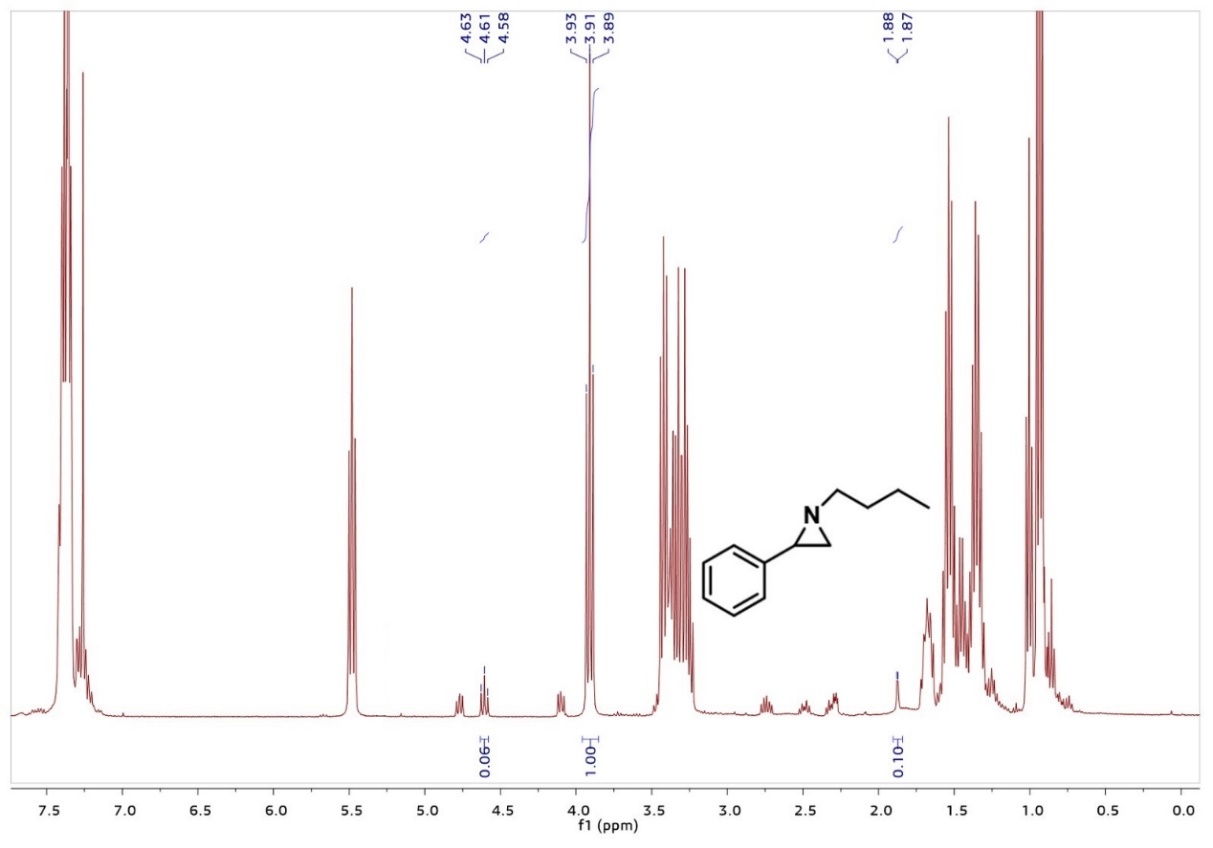


**Figure S26**. ^1^H NMR (400 MHz, CDCl_3_) spectrum of reaction system in Figure 4g entry 3. Conversion=(1+0.06)/(1+0.06+0.10)×100%=91.4%.


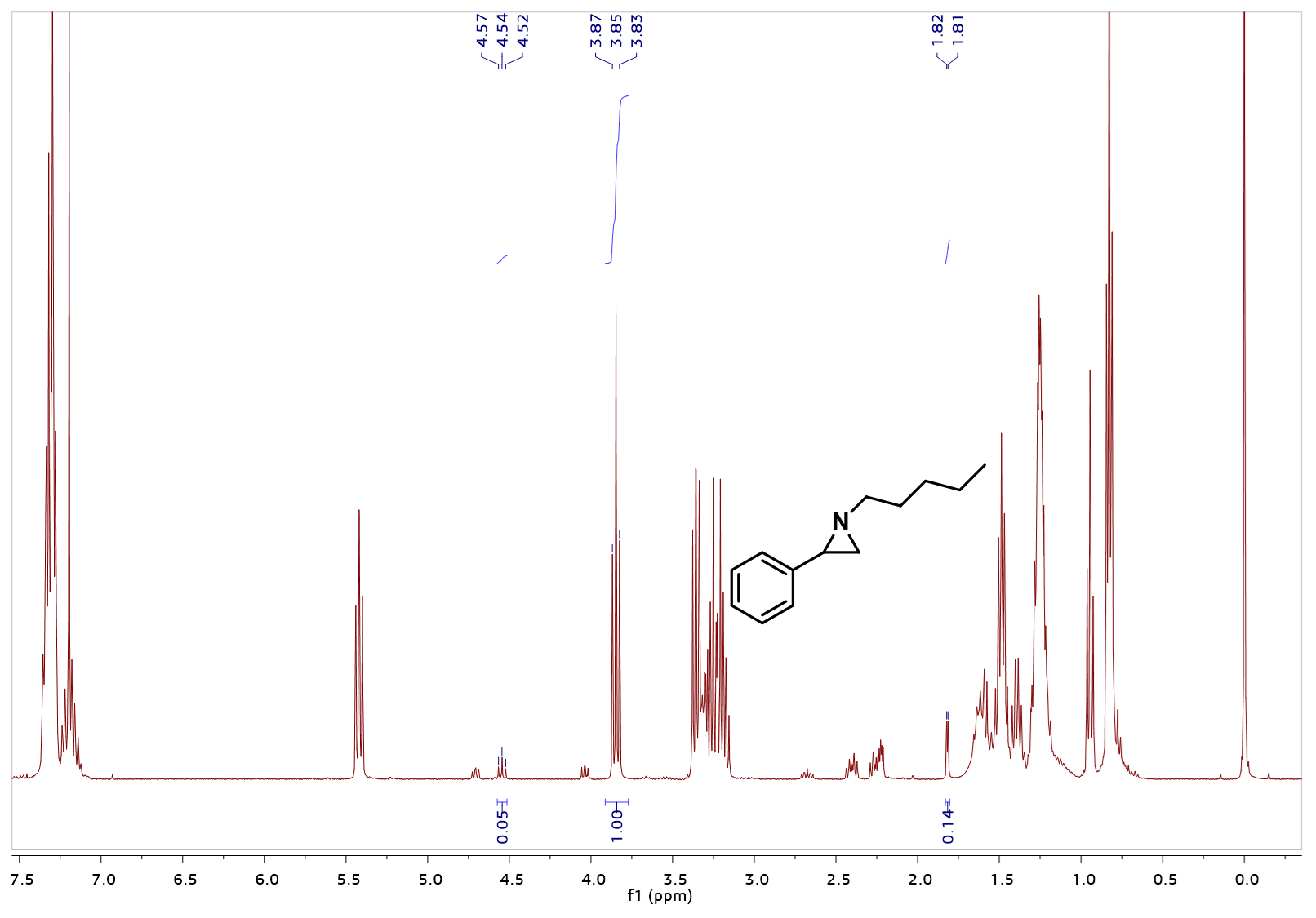


**Figure S27** ^1^H NMR (400 MHz, CDCl_3_) spectrum of reaction system in Figure 4g entry 4. Conversion=(1+0.05)/(1+0.05+0.14)×100 %=88.2%.


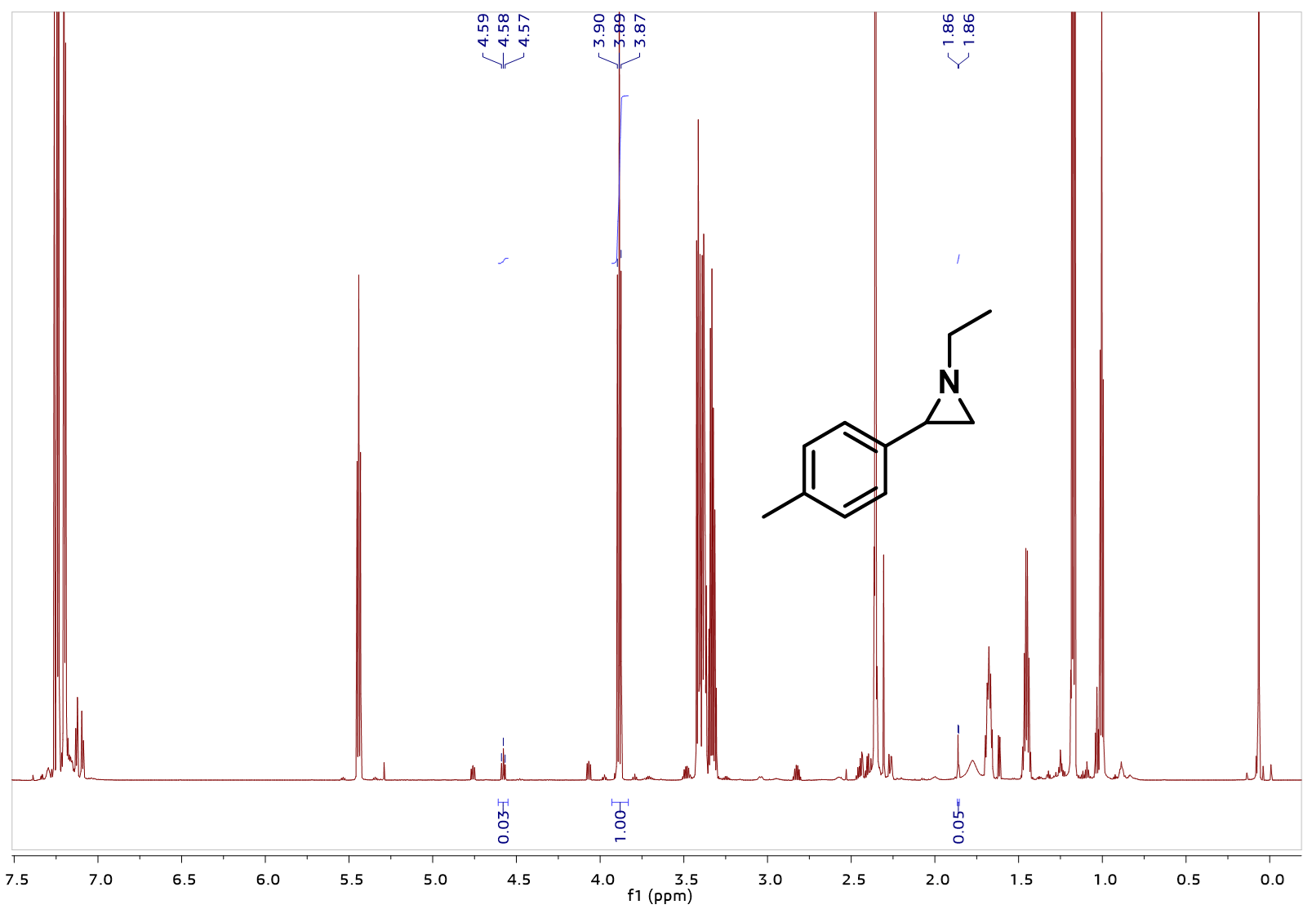


**Figure S28** ^1^H NMR (400 MHz, CDCl_3_) spectrum of reaction system in Figure 4g entry 5. Conversion=(1+0.03)/(1+0.03+0.05)×100 %=95.4%.


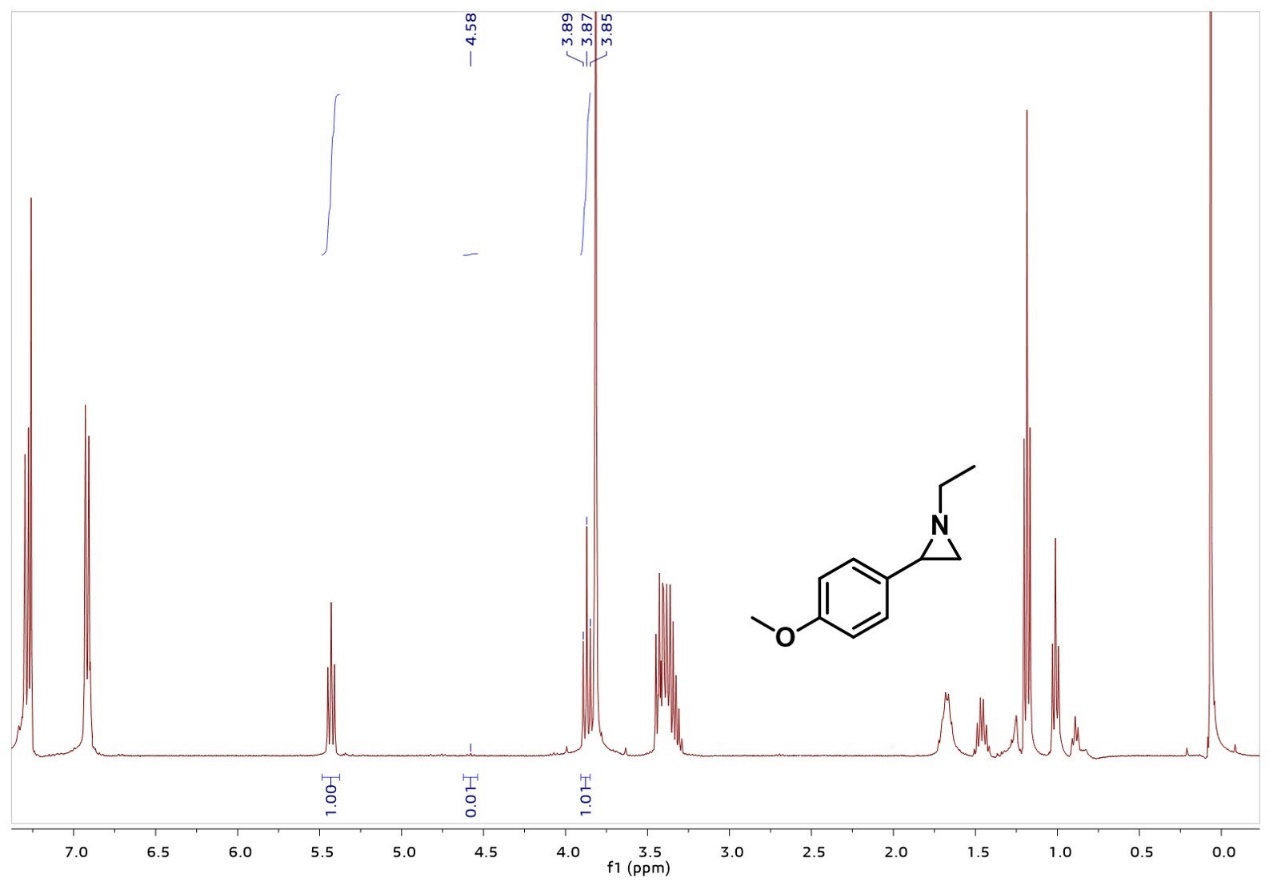


**Figure S29** ^1^H NMR (400 MHz, CDCl_3_) spectrum of reaction system in Figure 4g entry 6. Conversion>99%.


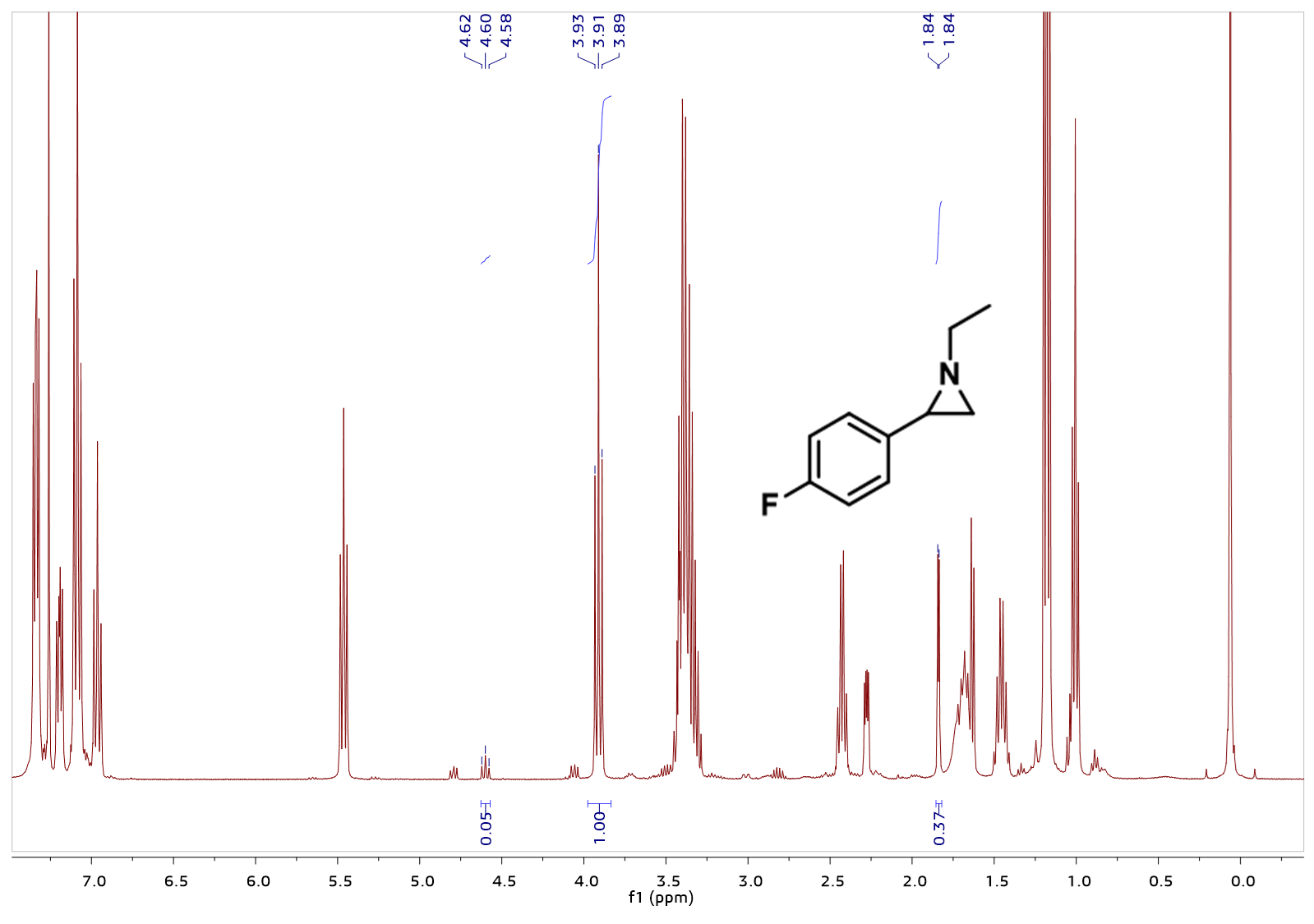


**Figure S30**. ^1^H NMR (400 MHz, CDCl_3_) spectrum of reaction system in Figure 4g entry 7. Conversion=(1+0.05)/(1+0.05+0.37)×100%=73.9%.


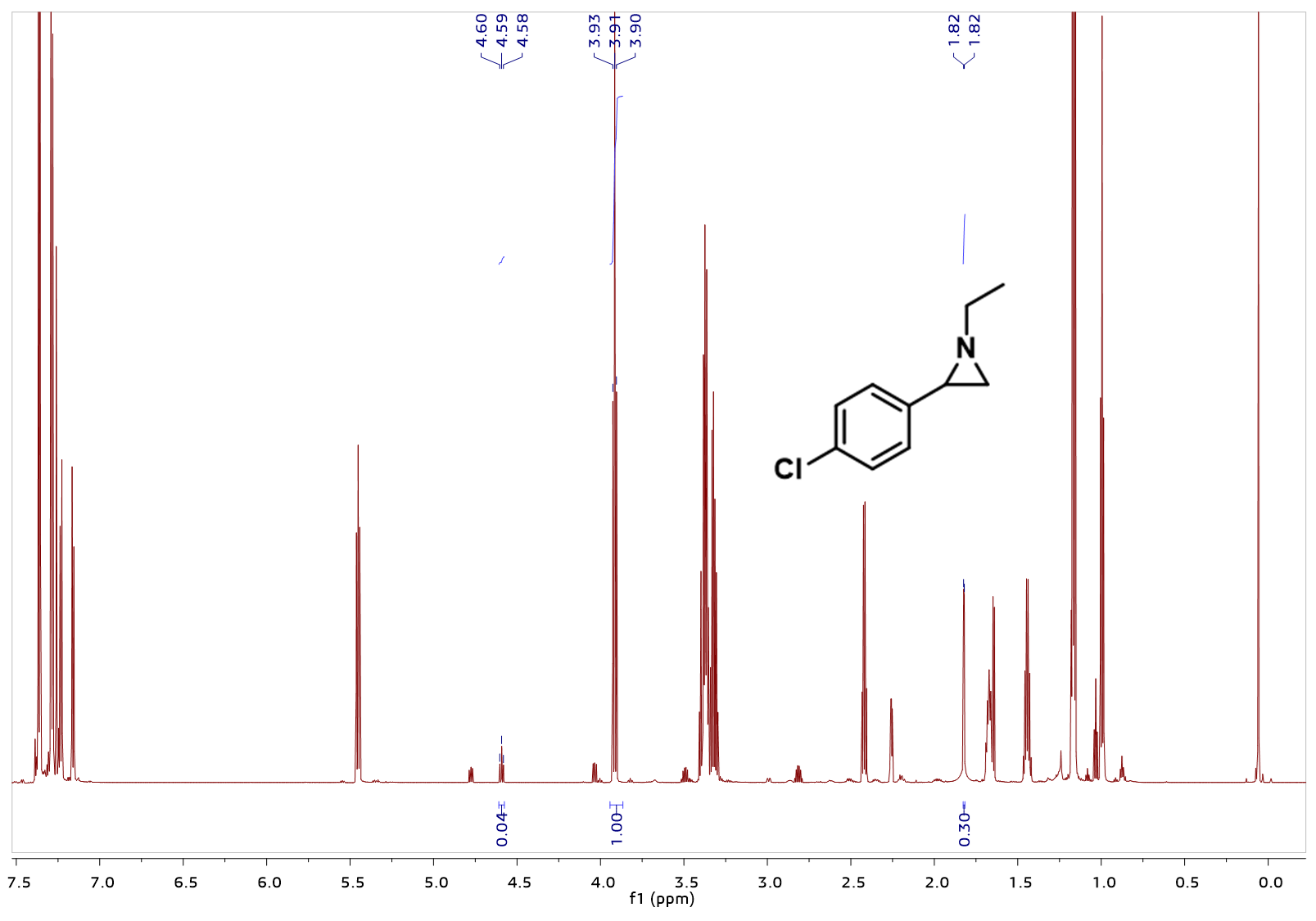


**Figure S31**. ^1^H NMR (400 MHz, CDCl_3_) spectrum of reaction system in Figure 4g entry 8. Conversion=(1+0.04)/(1+0.04+0.30)×100%=77.6%.

~~
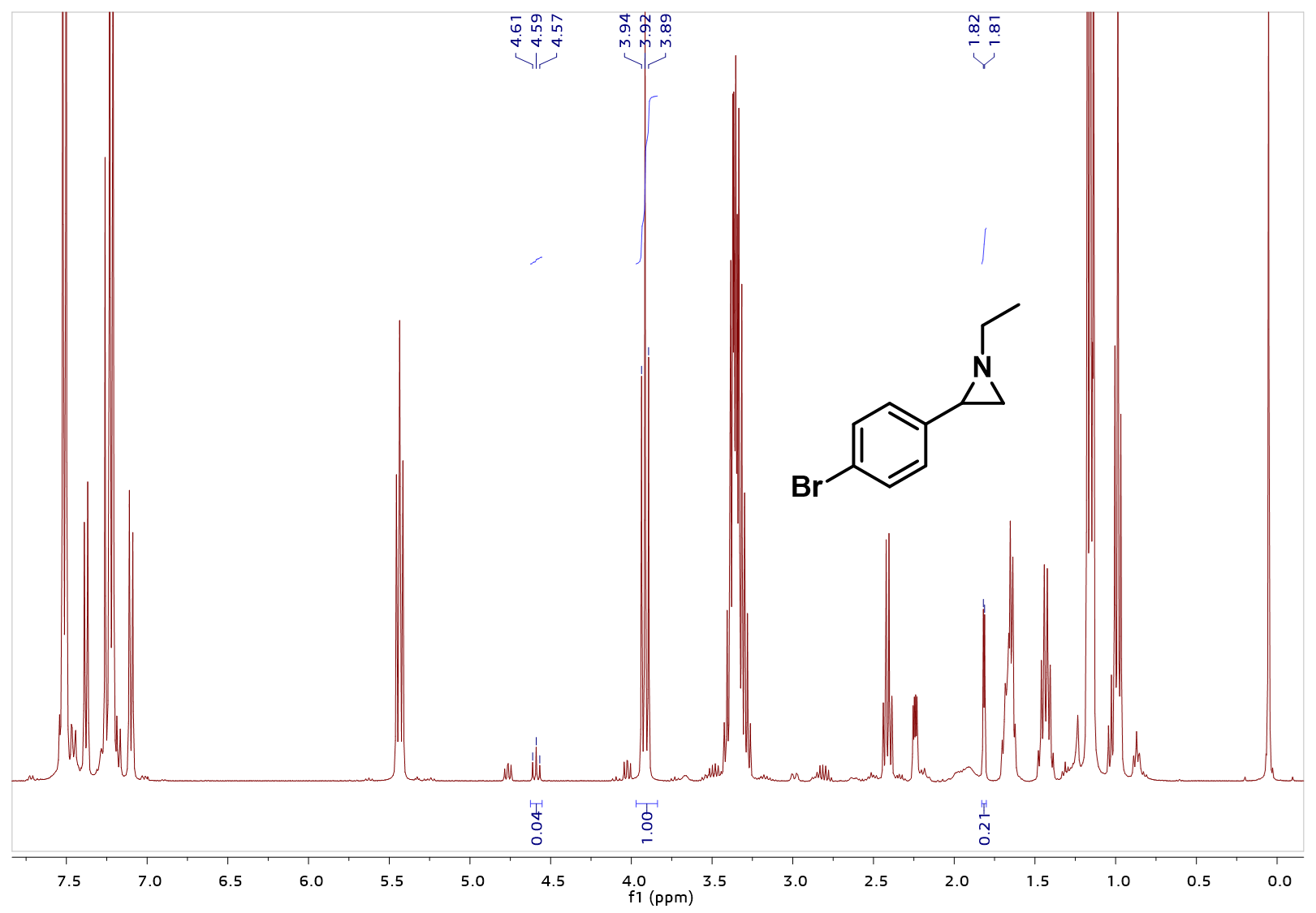
~~

**Figure S32** ^1^H NMR (400 MHz, CDCl_3_) spectrum of reaction system in Figure 4g entry 9. Conversion=(1+0.04)/(1+0.04+0.21)×100%=83.2%.

**Table S7** Comparison of catalytic CO_2_ cycloaddition activities for typical catalysts.

| **Catalysis type** | **Entry** | **Catalyst**  **/mg** | **Condition** | | | | | | **Conversion/%** | **Regio-sel** | **Reaction rate**  **/mol mol^-1^ h^-1^** | **Ref** |
| --- | --- | --- | --- | --- | --- | --- | --- | --- | --- | --- | --- | --- |
|  |  |  | **Substrate**  **/mmol** | **Metal ion center**  **/mol%** | **Cocatalyst**  **/mol%** | **T/****℃** | **P/**  **atm** | **Time/h** |  |  |  |  |
| Thermal-driven | 1 | Cu-MOF  80 mg | 2.0 | 10 | 5 | 100 | 20 | 12 | >99 | 98:2 | 0.83 | [3] |
|  | 2 | Zn-MOF1  20 mg | 2.0 | 2.8 | 5 | 70 | 20 | 10 | >99 | 98:2 | 3.54 | [4] |
|  | 3 | Co-MOF  20mg | 2.0 | 3.63 | 5 | 30 | 10 | 10 | 89 | 98:2 | 2.45 | [5] |
|  | 4 | Zn-MOF2  20mg | 2.0 | 5.3 | 5 | 70 | 20 | 12 | >99 | 98:2 | 1.56 | [6] |
|  | 5 | In-MOF  25mg | 2.0 | 1.7 | 5 | 30 | 10 | 10 | >99 | 99:1 | 5.82 | [7] |
|  | 6 | Ce-MOF  10mg | 1.0 | 1.4 | 3 | 70 | 5 | 12 | 97 | - | 5.77 | [8] |
|  | 7 | TPPH_2_ | aziridine in THF | 1.0 | 5 | 125 | 6 | 6 | >99 | 95:5 | 16.5 | [9] |
| Photo-driven | 8 | *m*-NiDBPA-COF  7.2mg | 2.0 | 0.5 | 5 | 30 | 1 | 48 | 99 | 96.2:3.8 | 4.13 | this work |

**6. References**

[1] Q. Shi, M.-H. Chen, J. Xiong, T. Li, Y.-Q. Feng, B. Zhang, “Porphyrin-Based Two-Dimensional Metal-Organic framework nanosheets for efficient catalytic CO_2_ transformation” *Chem. Eng. J.* **2024**, *481*, 148301.

[2] X. M. Kang, L. H. Yao, Z. H. Jiao, B. Zhao, “Two Stable Heterometal-MOFs as Highly Efficient and Recyclable Catalysts in the CO_2_ Coupling Reaction with Aziridines” *Chem. Asian J.* **2019**, *14*, 3668-3674.

[3] H. Xu, X. F. Liu, C. S. Cao, “A porous metal-organic framework assembled by [Cu_30_] nanocages: serving as recyclable catalysts for CO_2_ fixation with aziridines” *Adv. Sci.* **2016**, *3*, 1600048.

[4] C.-S. Cao, Y. Shi, H. Xu, B. Zhao, “A multifunctional MOF as a recyclable catalyst for the fixation of CO_2_ with aziridines or epoxides and as a luminescent probe of Cr(VI)” *Dalton Trans.* **2018**, *47*, 4545.

[5] X. M. Kang, Y.Shi, C. S. Cao, B. Zhao, “Stable metal-organic frameworks with high catalytic performance in the cycloaddition of CO_2_ with aziridines” *Sci. China Chem.* **2019**, *62*, 622-628.

[6] C. S. Cao, Y. Shi, H. Xu, B. Zhao, “An uncommon multicentered Zn^I^–Zn^I^ bond-based MOF for CO_2_ fixation with aziridines/epoxides” *Chem. Commun.* **2021**, *57*, 7537-7540.

[7] X. R. Tian, Y. Shi, S. L. Hou, “Efficient cycloaddition of CO_2_ and aziridines activated by a quadruple-interpenetrated indium-organic framework as a recyclable catalyst” *Inorg. Chem.* **2021**, *60*, 15383-15389.

[8] C. H. Zhang, Z. L. Wu, R. X. Bai, T. D. Hu, B. Zhao, “Highly Efficient Conversion of Aziridines and CO_2_ Catalyzed by Microporous [Cu_12_] Nanocages” *Acs Appl. Mater. Interfaces* **2023**, *15*, 1879-1890.

[9] C. Damiano, P. Sonzini, M. Cavalleri, G. Manca, E. Gallo, “The CO_2_ cycloaddition to epoxides and aziridines promoted by porphyrin-based catalysts” *Inorg. Chim. Acta* **2022**, *540*, 121065.
